# Maternal-derived galectin-1 shapes the placenta niche through Sda terminal glycosylation: Implication for preeclampsia

**DOI:** 10.1093/pnasnexus/pgad247

**Published:** 2023-08-01

**Authors:** Yiran Xie, Fangqi Zhao, Nancy Freitag, Sophia Borowski, Yiru Wang, Charlotte Harms, Poh-Choo Pang, Juliette Desforges, Tianyu Wen, Edzard Schwedhelm, Manvendra Singh, Ralf Dechend, Anne Dell, Stuart M Haslam, Gabriela Dveksler, Mariana G Garcia, Sandra M Blois

**Affiliations:** Department of Obstetrics and Fetal Medicine, University Medical Center Hamburg-Eppendorf, 20251 Hamburg, Germany; Department of Obstetrics and Fetal Medicine, University Medical Center Hamburg-Eppendorf, 20251 Hamburg, Germany; Department of Obstetrics and Fetal Medicine, University Medical Center Hamburg-Eppendorf, 20251 Hamburg, Germany; Experimental and Clinical Research Center (ECRC), a cooperation of the Max-Delbrück Center for Molecular Medicine (MDC) and Charité-Universitätsmedizin, 13125 Berlin, Germany; Department of Obstetrics and Fetal Medicine, University Medical Center Hamburg-Eppendorf, 20251 Hamburg, Germany; Experimental and Clinical Research Center (ECRC), a cooperation of the Max-Delbrück Center for Molecular Medicine (MDC) and Charité-Universitätsmedizin, 13125 Berlin, Germany; Department of Obstetrics and Fetal Medicine, University Medical Center Hamburg-Eppendorf, 20251 Hamburg, Germany; Department of Obstetrics and Fetal Medicine, University Medical Center Hamburg-Eppendorf, 20251 Hamburg, Germany; Department of Life Sciences, Imperial College London, London SW7 2AZ, UK; Department of Life Sciences, Imperial College London, London SW7 2AZ, UK; Department of Life Sciences, Imperial College London, London SW7 2AZ, UK; Institute of Clinical Pharmacology and Toxicology, University Medical Center Hamburg-Eppendorf and German Centre for Cardiovascular Research (DZHK), Partner Site Hamburg/Kiel/Lübeck, 20249 Hamburg, Germany; Clinical Neuroscience, Max Planck Institute for Multidisciplinary Sciences, 37075 Göttingen, Germany; Experimental and Clinical Research Center (ECRC), a cooperation of the Max-Delbrück Center for Molecular Medicine (MDC) and Charité-Universitätsmedizin, 13125 Berlin, Germany; Department of Cardiology and Nephrology, HELIOS-Klinikum, 13125 Berlin, Germany; Department of Life Sciences, Imperial College London, London SW7 2AZ, UK; Department of Life Sciences, Imperial College London, London SW7 2AZ, UK; Department of Pathology, Uniformed Services University of the Health Sciences, Bethesda, MD 20814, USA; Department of Obstetrics and Fetal Medicine, University Medical Center Hamburg-Eppendorf, 20251 Hamburg, Germany; Department of Obstetrics and Fetal Medicine, University Medical Center Hamburg-Eppendorf, 20251 Hamburg, Germany

**Keywords:** galectin-1, maternal niche, preeclampsia, Sda antigen

## Abstract

Placental abnormalities cause impaired fetal growth and poor pregnancy outcome (e.g. preeclampsia [PE]) with long-lasting consequences for the mother and offspring. The molecular dialogue between the maternal niche and the developing placenta is critical for the function of this organ. Galectin-1 (gal-1), a highly expressed glycan-binding protein at the maternal–fetal interface, orchestrates the maternal adaptation to pregnancy and placenta development. Down-regulation or deficiency of gal-1 during pregnancy is associated with the development of PE; however, the maternal- and placental-derived gal-1 contributions to the disease onset are largely unknown. We demonstrate that lack of gal-1 imposes a risk for PE development in a niche-specific manner, and this is accompanied by a placental dysfunction highly influenced by the absence of maternal-derived gal-1. Notably, differential placental glycosylation through the Sda-capped N-glycans dominates the invasive trophoblast capacity triggered by maternal-derived gal-1. Our findings show that gal-1 derived from the maternal niche is essential for healthy placenta development and indicate that impairment of the gal-1 signaling pathway within the maternal niche could be a molecular cause for maternal cardiovascular maladaptation during pregnancy.

**Significance Statement**

The placenta is formed from the molecular dialogue within the maternal niche; failure in placentation increases the risk of developing preeclampsia (PE), a life-threatening disease associated with lifelong adverse health consequences for the mother and offspring. Mechanisms underlying the maternal contribution to the placentation process remain unclear. This work uncovered the critical role of maternal galectin-1 (gal-1) in mammalian reproduction. Using specific niche *Lgals1*-deficient models, we determined that maternal-derived gal-1 boosts the invasive trophoblast capacity by modulating differential Sda terminal N-glycosylation and HB-EGF bioavailability at the maternal–fetal interface. These observations provide a better understanding of the maternal molecular dialogue involved in the placental adaptations required for a successful pregnancy.

## Introduction

Preeclampsia (PE) is a complication of pregnancy defined by hypertension with or without proteinuria developed after the 20th week of gestation. This heterogeneous syndrome is a leading cause of maternal and neonatal mortality and morbidity worldwide (1). Due to the increased prenatal surveillance and early interventions, the mortality of PE has significantly dropped in several countries, including the United States. Nevertheless, the rising burden of PE short- and long-term cardiovascular sequelae impacts both maternal and child health (2, 3). The etiology of PE continues to be debated; however, research in this field has significantly contributed to understanding the disease pathogenesis, where the placenta component plays a central role (4, 5). Delivery of the placenta and the usually preterm baby remains the only available treatment; therefore, deciphering the underlying mechanisms of the disease is of great importance.

The placenta forms the maternal–fetal interface and determines fetal growth and survival supplying oxygen, nutrients, and waste exchange. Thus, impaired placenta function is generally associated with poor fetal outcomes and increased shedding of trophoblast microparticles into the maternal circulation (4). The inflammatory and antiangiogenic placenta shedding causes maternal endothelial cell activation, a hallmark of PE, and is responsible for the disease's clinical symptoms (5, 6). The role of the placenta as the trigger for the maternal syndrome has been extensively studied in the PE field (4, 7). However, the contribution of the maternal niche to the etiology of PE remains in question. Studies in humans profiling the chorionic villus have shown that impaired decidua maturation is associated with the development of PE (8). Moreover, human endometrial stromal cells isolated from women with severe PE failed to decidualize in vitro and did not support cytotrophoblast (CTB) invasion (9). These observations suggest that defects in the maternal niche could be a critical contributor to the placenta maladaptation triggering PE disease by diverse molecular pathways (10). However, molecular mechanisms governing the maternal–placental dialogue remain to be elucidated.

Previous studies by our and other groups showed that galectin-1 (gal-1), a member of the conserved family of soluble β-galactoside-binding proteins, is the most abundant galectin at the maternal–fetal interface (11–14). Gal-1 is a key regulator of the placental–maternal dialogue due to its ability to regulate critical processes, including maternal immune and vascular adaptation to pregnancy and trophoblast differentiation and invasion properties (13, 15–17). Indeed, gal-1-deficient pregnant dams developed a spontaneous PE-like syndrome characterized by hypertension, proteinuria, and fetal growth restriction (FGR) (17). Inhibition of endogenous gal-1 decreased the invasive capacity of human extravillous trophoblast affecting the spiral artery (SpA) remodeling process (16). Moreover, a decrease in circulating gal-1 levels between 8 and 10 weeks of gestation was observed in women who later developed PE (17). A reduced frequency of gal-1 expressing T and natural killer (NK) cells was reported in the peripheral blood of women diagnosed with PE, suggesting a role of this galectin in the development of PE (18). To gain further insight into the gal-1 maternal and placental contribution through which PE develops, we undertook an experimental in vivo model in which lack of gal-1 is conferred exclusively to the maternal or the placental niche (19). Here, we demonstrated that (i) gal-1 expression is higher in the maternal niche than the placental compartment during the period, (ii) gal-1 deficiency within the maternal niche sensitizes the cardiovascular adaptation to pregnancy and induces a PE-like syndrome, (iii) lack of maternal gal-1 signaling leads to impaired placenta function characterized by a distinguished glycopattern (e.g. loss of Sda-capped N-glycans) before PE onset, and (iv) maternal-derived gal-1 is a critical regulator of the heparin-binding epidermal growth factor-like growth factor (HB-EGF)/β-1,4-N-acetylgalactosaminyltransferase 2 (B4GALNT2) loop responsible for the synthesis of Sda-capped glycans on invasive trophoblasts. We propose that the maternal niche, through gal-1, is a pivotal contributor to healthy placenta function and, therefore, to the etiology of PE. Therapies designed to improve gal-1 expression in the maternal niche could be a novel strategy to prevent placenta pathologies with poor pregnancy outcomes.

## Results

### Maternal gal-1 deficiency sensitizes cardiovascular adaptation imposing a risk for PE development

**Fig. 1.**
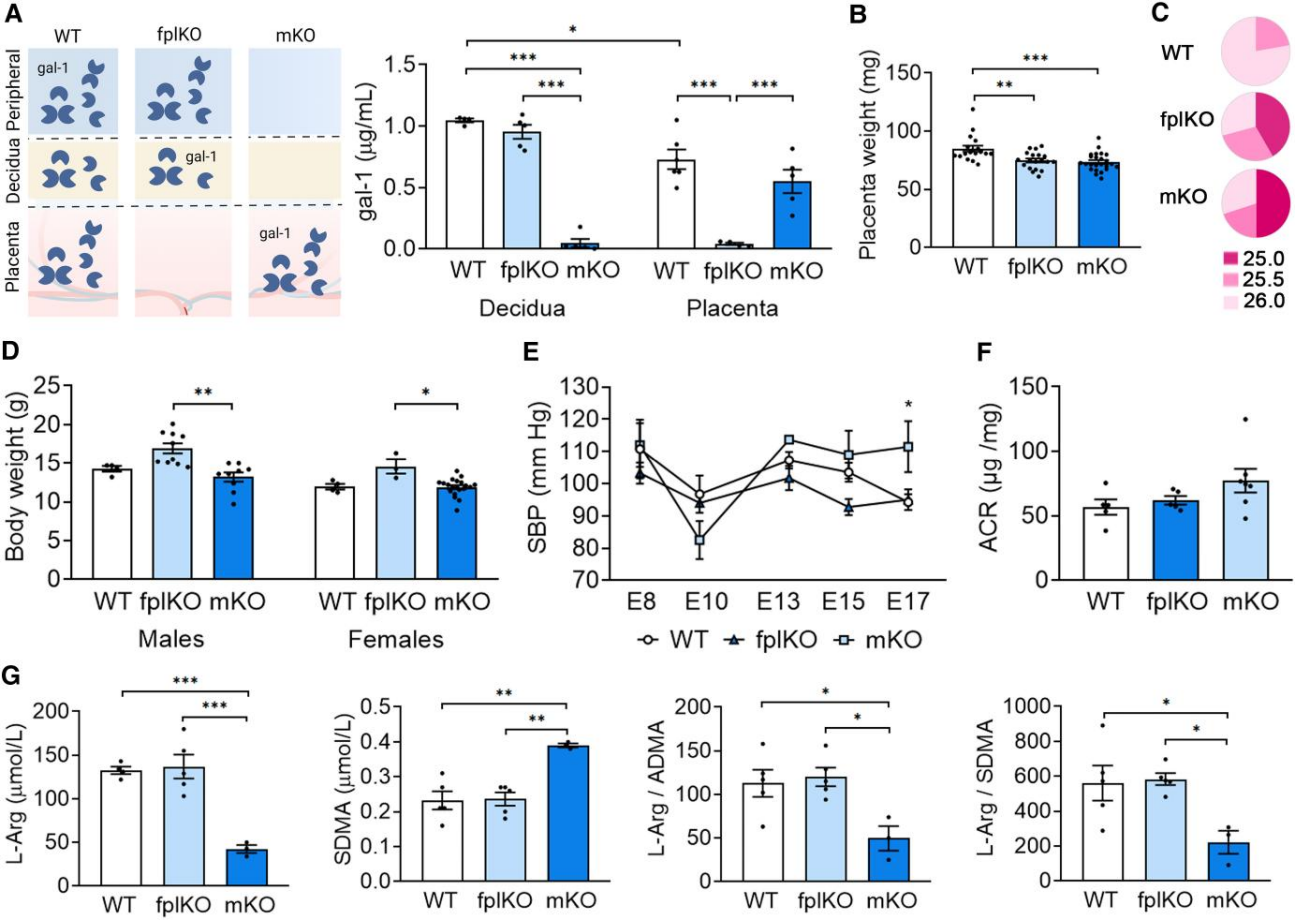
Deficiency of maternal gal-1 induces cardiovascular maladaptation in mice. A) Schematic depiction of gal-1 expression at the maternal–fetal interface and periphery in the experimental groups: *Lgals1^+/+^* (WT), fplKO and mKO *(left)*. Gal-1 levels measured by ELISA in placenta and decidua tissue homogenates on embryonic day E13 (*n* = 6) (right). B) Placental weight (mg) was registered on E17. C) Fetal development defined by Theiler stage (TS) analysis was evaluated on fetuses derived from WT, fplKO, and mKO on E17. In mKO and fplKO pregnancies, higher proportion of fetuses were on TS 25 (*P* < 0.01 vs. WT, chi-square test), and lesser proportion reached the TS 26 (*P* < 0.01 vs. WT, chi-square test). D) Offspring body weight (grams) was registered on PN day 28. E) Systolic blood pressure (SBP) was measured from E8 to E17 (*n* = 5) by a noninvasive tail-cuff acquisition system (CODA System). F) Ratio of albumin to creatinine (ACR) in urine collected on E17 (*n* = 6). G) Measurement of L-Arg, SDMA, and the ratio of L-Arg/ADMA and L-Arg/SDMA in maternal circulation at E13 (*n* = 5) by MS/MS assay. In all figures, data are presented as the mean ± SEM. In A) and G), *P < 0.05, **P < 0.01, and ***P < 0.001 using one-way ANOVA followed by Tukey's test; in E), *P < 0.05 using two-way ANOVA followed by Tukey's test, and in B) and D), *P < 0.05, **P < 0.01, and ***P < 0.001 using Kruskal–Wallis followed by Dunn's test.

We first analyzed the expression of gal-1 at the maternal and placental compartments during normal pregnancy. As shown in Fig. 1A, the levels of gal-1 are higher in the maternal niche compared with the placenta compartment at embryonic day (E)13 in wild-type (WT) dams. As experimental models to understand the importance of gal-1 in each compartment, we used in vitro fertilization to achieve either maternal gal-1 deficiency (mKO) or fetal–placental gal-1 deficiency (fplKO) (19). Lack of gal-1 expression within the decidua (mKO) and placenta (fplKO), respectively, are displayed in Fig. 1A. Previously, we showed that E13 fetuses carried by mKO dams suffered from FGR and fetal immaturity when compared with fplKO and WT dams (19). Here, we extended the analysis to the late gestation (E17) and postnatal (PN)28 period. We observed that placenta weights were lower and fetuses carried by fplKO and mKO dams displayed fetal immaturity (as shown by the lower Theiler stage distribution at E17) when compared with the WT dams (Fig. 1B and C). In addition, pups delivered by mKO dams showed significantly lower body weight when compared with fplKO mice on PN28 (Fig. 1D), suggesting that the altered fetal growth trajectory may affect the PN development. Alongside the observed FGR phenotype at E13, maternal systolic blood pressure was increased from E13 in mKO dams and was significantly higher compared with the fplKO and control dams at E17 (Fig. 1E). On the other hand, mKO displayed an increased urine albumin/creatinine ratio (ACR), but this was not statistically different when compared with fplKO and WT dams on E17 (Fig. 1F). Because hypertension is a clinical sign originating from endothelial dysfunction (1), we next characterized the cardiometric adaptation before disease onset. A normal endothelial function requires nitric oxide (NO) production from L-arginine (L-Arg). L-Arg can be methylated, and one of its derivatives, asymmetric dimethylarginine (ADMA), inhibits endothelial NO synthase (eNOS) activity (20). Elevated ADMA serum levels are associated with reduced vasodilation and endothelial dysfunction, and the ratio of L-Arg to ADMA is a valuable index to evaluate eNOS activity (21). Another L-Arg derivative, symmetric dimethylarginine (SDMA), also modifies endothelial function by competition with L-Arg transport without affecting eNOS activity (22). As shown in Fig. 1G, the L-Arg concentration is decreased, while the SDMA levels are increased in the maternal circulation of mKO compared with the WT. Furthermore, the ratio of L-Arg/ADMA and L-Arg/SDMA is decreased in mKO, reflecting limited NO production. These findings suggest that gal-1 derived from the maternal niche is important for cardiovascular adaptation during pregnancy and impacts fetal maturity preceding the onset of maternal PE syndrome.

### Lack of maternal-derived gal-1 has a significant impact on the placentation process

To further examine the effects of maternal-derived gal-1 at the maternal–fetal interface, we analyzed the post-placentation period (E13) prior to PE onset. The mature murine placenta consists of three layers: the labyrinth (Lab), the junctional zone (Jz), and the maternal decidua basalis (DB) (Fig. S1A). As shown in Fig. S1B, we observed a reduction in the decidua area in mKO with less mature (DBA^+^PAS^+^) tissue-associated uterine NK cells (Fig. S1C). However, inflammation and areas of necrosis were apparent in the decidua area of both mKO and fplKO dams (Fig. S1D). Uterine vasculature transformation is a unique process that depends on uNK cells and invasive trophoblasts (4). Strikingly, SpA wall thickness (vessel/lumen ratio) (Fig. S1E), the number of mature vascular-associated uNK cells (Fig. S1E), and pan-cytokeratin^+^ trophoblast cells were reduced in both experimental models (Fig. S1E), suggesting that the SpA remodeling is influenced by the gal-1 derived from both niches. SpA remodeling is typically associated with the loss of pericytes (α-SMA positive cells) (6). We observed that the mKO decidua displayed an increased number of pericytes compared with WT and fplKO dams (Fig. S1F), indicating more severely impaired vascular remodeling in mice lacking gal-1 in the maternal compartment.

Further analysis revealed that the number of glycogen cells (GCs) in the Jz (PAS^+^ cells/mm^2^) is increased in fplKO, and mislocalized GC islets within the Lab layer of the placenta are evident in both fplKO and mKO mice (Fig. S2A). The Lab layer of the placenta is the site of nutrient exchange, and any defect may influence the ability to mediate the transfer of nutrients and oxygen to the fetus thus impacting fetal growth (23). Quantitative analysis of the fetal vasculature of the Lab (isolectin B4^+^) showed an increase in the total vessel area and branching, resulting in a reduced lacunarity in mKO dams (Fig. S2B). Interestingly, the fplKO Lab showed slightly reduced vasculature complexity (decreased area occupied by vessels, normal branching, and lacunarity). The placenta insufficiency phenotype at E13 is accompanied by an inflammatory trophoblast state (Fig. S2C). Thus, the augmented placenta inflammation in mKO dams suggests a decisive contribution of the maternal-derived gal-1 to the placentation process.

Since the feto-maternal interface simultaneously expresses several galectins, we posited that in the gal-1 knockout models, there could be functional compensation by other galectin family members. Therefore, to determine the galectin fingerprint in gal-1 knockout models, we used multiplex imaging to visualize the three most abundant galectins (gal-1, gal-3, and gal-9) in the post-placentation period (E13) (Fig. S2D). We observed that while in WT mice, uNK cells/decidual stromal cells expressed weak/moderate levels of gal-3, respectively, the levels were higher in mKO and fplKO within the same cellular compartments. Analysis of gal-9 expression demonstrated a similar nuclear localization in all experimental groups (Fig. S2D). Concerning the trophoblasts that invaded the maternal decidua, mKO and fplKO showed a marked increase of gal-3 expression compared with the WT. Nuclear gal-9 expression was moderate in all groups and colocalized with gal-3. We conclude that mKO and fplKO compensated for the lack of gal-1 with gal-3 and gal-9 within the decidual compartment. However, distinct phenotypes in mKO and fplKO support the functional uniqueness role of gal-1 in the maternal niche.

Defective decidualization is known to influence the development of PE in humans (9); therefore, we next assessed the maternal vascular expansion on the pre-placentation period (E7) (Fig. S3A). Quantitative analysis of the vascular tree of the whole implantation site (endoglin^+^ vessels) showed a decrease in the total vessel area and of the total vessel length, resulting in a reduced vascular expansion in mKO dams when compared with WT dams (Fig. S3B). Comparison of the decidualization translational genes revealed a significant decrease of *Prl8a2* and *Wnt5a* decidual expression in mKO dams compared with WT (Fig. S3C). This was accompanied by an aberrant uNK activation (degranulated perforin^+^ NK cells) in the pre-placentation period (Fig. S3D). These results suggest that lack of maternal gal-1 during decidualization caused inadequate vascular expansion, differential NK activation, and decrease in decidualization makers which may influence trophoblast invasion into the decidua.

### Loss of placental Sda-capped glycans anticipates the development of PE-like syndrome

**Fig. 2.**
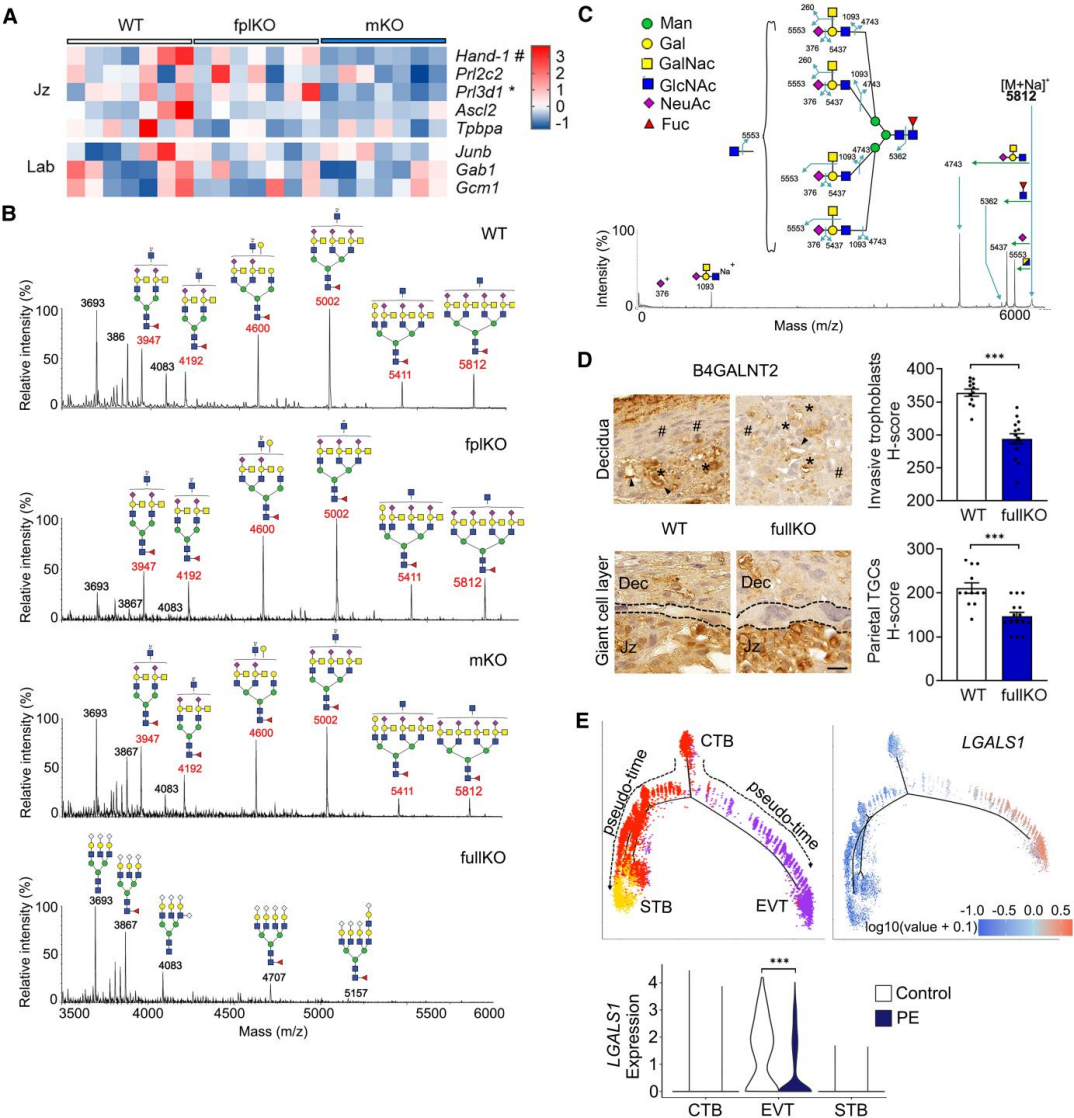
Absence of placental Sda-capped glycans is associated to gal-1 deficiency. A) Heatmap showing the expression of trophoblast developmental genes specific for the Jz and Lab layers, analyzed by qPCR followed by *Z*-score normalization (*n* = 7), ^#^*P* < 0.05 mKO vs. WT and fplKO; *P < 0.05 mKO vs. WT using Kruskal–Wallis followed by Dunn's test. B) MALDI-TOF MS spectra of N-glycans in the high mass range (m/z 3,500–6,000) extracted from bulk placental tissue derived from WT, fplKO, mKO, and fullKO dams on embryonic day E13. C) MALDI-TOF/TOF MS/MS fragmentation analysis of [M + Na] + peak m/z 5,812, illustrating the loss of Sda antigen. Man, mannose; Gal, galactose; GalNAc, N-acetylgalactosamine; GlcNAc, N-acetylglucosamine; NeuAc, N-acetylneuraminic acid; Fuc, Fucose. D) Representative images of B4GALNT2 immunohistochemistry staining within the decidua compartment (upper) and giant cell layer (lower) of the placenta on E13. Scale bar: 20 µm. In the decidua, uterine NK cells (^#^), invasive trophoblasts (asterisk), and endothelial cell (arrow) are shown. The giant cell layer (delineated by dashed lines) is located between the decidua (Dec) and Jz. Quantification of the expression of B4GALNT2 on invasive trophoblast (upper right) or parietal trophoblast giant cells (TGC, lower right) expressed as *H*-score, ***P < 0.001 using two-tailed *t* test. E) Pseudotime trajectory showing the ordering of major trophoblast lineages at the maternal–fetal interface (upper (left)) was performed from scRNA-seq data set analysis. Monocle2 visualization of three distinct clusters of trophoblasts using the top 2,000 differentially expressed genes projected into a 2D space. Codes represent CTB (middle), syncytiotrophoblast (STB, left), and extravillous trophoblast (EVT, right). Top right panel illustrates intensity and abundance of *LGALS1* transcript expression along the trajectory in previous figure. Colors represent an average Log2 expression level scaled to the number of unique molecular identification (UMI) values in single cells. The color scale is from blue to red, corresponding to lower to higher expression, respectively. Expression of *LGALS1* transcript (lower (left)) in the healthy and preeclampsia trophoblast cell types, viz. CTB, EVT, and STB ***P < 0.001 using Wilcoxon rank-sum test. fullKO, *Lgals1^−/−^*; WT, *Lgals1*^+/+^.

Placenta insufficiency can result from delayed trophoblast differentiation (24). To determine whether the lack of maternal-derived gal-1 could impact the differentiation stage of the trophoblast subsets, we assessed the gene expression profile that characterized the Jz and Lab layers of the placenta at E13 before PE onset. mKO-derived placentas showed a reduction of *Hand-1*, an essential transcription factor for trophoblast giant cell differentiation, and of placental lactogen I (*Prl3d1*), which is expressed by terminally differentiated trophoblast giant cells within the Jz compared with WT and fplKO-derived placentas (Fig. 2A). This indicates that impaired maternal-derived gal-1 signaling delayed placenta differentiation of the Jz. However, we observed similar levels of expression of genes involved in Lab development.

Glycans modify proteins required for trophoblast function, and previous studies showed that altered glycosylation is associated with placental insufficiency (25–27). To determine whether the placenta glycophenotype differed in the mKO dams at E13, we used a MALDI-MS/MS-based glycomic approach (28, 29) to define the N-glycan profiles of placental tissue from WT, fplKO, mKO, and fullKO mice. All samples contained similar N-glycome profiles in the low to mid mass range (m/z 1,500–3,500), where high mannose and biantennary complex-type N-glycans are abundant (Figs. 2B and S4). However, clear differences were observed in the high mass range (m/z 3,500–6,000) in the absence of gal-1, as illustrated in Fig. 2B. Comparable glycomic profiles were found in three biological replicates (Figs. S4 and S5). The WT, fplKO and mKO spectra are characterized by a molecular ion series at m/z 3,947, 4,600, 5,002, 5,411, and 5,812. MS/MS experiments revealed that these components are complex, core-fucosylated tri- and tetra-antennary glycans with up to four antenna capped by the Sda epitope GalNAcβ1–4(ΝeuAcα2-3)Galβ1-4GlcNAc (Figs. 2B, S4, and S5). Additional antennae are either α-Gal capped or are truncated at GlcNAc (see cartoon annotations in Fig. 2C). Thus m/z 3,947 and m/z 4,600 have two Sda antennae, m/z 5,002 and 5,411 have three Sda antennae, and m/z 5,812 has four Sda antennae. A single α-Gal-capped antenna is found in the m/z 4,600 and m/z 5,812 glycans. The overall spectra for WT, fplKO, and mKO in this mass range closely resemble each other, although that of the mKO has somewhat reduced levels of the Sda-containing glycans compared with WT, while the Sda abundance in fplKO is higher (Figs. 2B, S4, and S5). In contrast, Sda-containing glycans are either absent or low in abundance in the fullKO. Instead, its glycome is dominated by NeuGc-capped glycans in the high mass range (Fig. 2B). Therefore, we conclude that gal-1 contributes significantly to Sda regulation since the absence of this lectin in the fullKO is associated with an almost complete loss of Sda epitopes. These results constitute additional evidence of the maternal-derived gal-1 contribution to placental differential glycosylation which antedates the development of PE.

The last step in the biosynthesis of Sda-capped glycans is catalyzed by B4GALNT2, and inhibition of this enzyme impaired mouse embryo implantation (30). As shown in Fig. 2D, expression of B4GALNT2 is down-regulated in the decidua and trophoblast GC layer of gal-1 fullKO placenta, suggesting that gal-1 may regulate B4GALNT2 expression on the invasive trophoblast lineages. Knowing that endogenous gal-1 also is involved in the invasive machinery of trophoblast (13, 15–17), we investigated a recently published single-cell RNA-sequencing (RNA-seq) analysis of placenta and decidua biopsies to explore a possible association of *LGALS1* expression levels with trophoblast differentiation trajectory in humans (31). To this end, we assessed the cells annotated as “trophoblast” from the original study and constructed their pseudotime trajectory using the “Monocle2” tool. Consistent with previous results (15, 17), extravillous CTBs (EVT) bifurcate from the trajectory of CTB to syncytiotrophoblasts (STB) and are placed on a single branch toward the end of the pseudotime trajectory of trophoblast (see Materials and Methods S1). This recapitulated the biological trajectory of trophoblast differentiation. Upon examining the *LGALS1* transcript expression, we observed that was abundantly and specifically expressed in the EVT lineage, while showing higher expression along the trajectory of EVT (Fig. 2E). Next, we asked if the trophoblast of women suffering from PE have differential expression in comparison with healthy counterparts. Indeed, EVT derived from PE women showed a significant down-regulation of *LGALS1* when compared with women that have a normal pregnancy (Fig. 2E). Overall, these results are consistent with our hypothesis that decidual gal-1 deregulation is associated with PE disease.

### Gal-1 facilitates trophoblast invasive properties through Sda-capped glycans and HB-EGF maturation

**Fig. 3.**
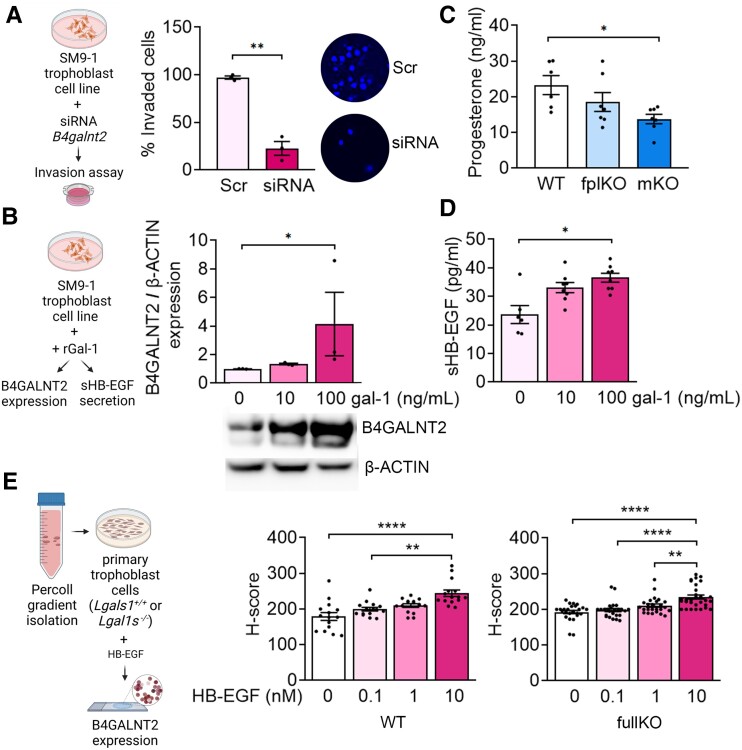
Exogenous gal-1 is involved in trophoblast invasion through Sda-capped glycans and sHB-EGF. A) Schematic drawing of murine trophoblast cell line SM9-1 culture condition with *B4galnt2* siRNA. The invasion capacity of SM9-1 cells was evaluated by transwell invasion assay (left). Percentage of invaded SM9-1 cells (middle) and representative images (right) of SM9-1 with siRNA for *B4galnt2* (siRNA) or a scramble sequence (Scr) as control (*n* = 3, 5 pictures/assay), **P < 0.01 using two-tailed *t* test. B) Experimental designs of the murine trophoblast cell line SM9-1 treatment with recombinant gal-1 (rGal-1) after 24-h B4GALNT2 expression and sHB-EGF levels in the culture supernatant were analyzed by ELISA. B4GALNT2 protein expressions were tested by western blot in SM9-1 cells upon stimulation with 0, 10, or 100 ng/mL of rgal-1 in three independent experiments. ß-ACTIN was used to calculate relative B4GALNT2 protein expression levels. C) Circulating progesterone levels measured on E13 in WT, fplKO, and mKO dams by ELISA (*n* = 7). D) sHB-EGF ELISA determination in the supernatant derived from SM9-1 cells stimulated with 0, 10, and 100 ng/mL of rgal-1 for 24 h (*n* = 6). E) Primary trophoblasts derived from *Lgals1^+/+^* (WT) or *Lgals1^−/−^* (fullKO) placentas on E13 were isolated by Percoll gradient, and B4GALNT2 expression was evaluated upon treatment with recombinant HB-EGF (0, 0.1, 1, or 10 nM HB-EGF) for 24 h using IHC. *H*-score was obtained from the analysis of three independent experiments (five images/experimental condition). In all figures, data are plotted as the mean ± SEM. In B)–E), *P < 0.05, **P < 0.01, ***P < 0.001, and ****P < 0.0001 using Kruskal–Wallis followed by Dunn's test.

To further characterize the role of Sda-capped glycans in pregnancy, we inhibited the B4GALNT2 enzyme by siRNA in the murine SM9–1 and human HTR-8/SVneo trophoblast cell lines (Figs. 3A and S6A and B, Table S1 and S2). Reduction of B4GALNT2 levels in the mouse SM9-1 and human HTR-8/SVneo trophoblast cell lines significantly impaired their invasion capacity (Fig. 3A and S6C). In addition, treatment of SM9-1 cells with exogenous gal-1 increased the expression of B4GALNT2 (Fig. 3B). These results indicated that gal-1 increased the activity of B4GALNT2 and that this enzyme is involved in the invasion capacity of trophoblast cells, a critical process necessary for the maternal vasculature transformation. To identify if progesterone, a known inducer of B4GALNT2 expression (30), was down-regulated in the gal-1-deficient pregnancies, we determined the maternal levels at E13. This analysis revealed that gal-1 deficiency in the maternal niche is associated with decreased progesterone in maternal circulation (Fig. 3C). Outside reproductive tissues, soluble heparin-binding epidermal growth factor-like growth factor (sHB-EGF) induces the expression of B4GALNT2 (32). Of note, HB-EGF is expressed at high levels and was associated with trophoblast cells’ survival and invasive capacity during the first trimester of pregnancy (33). Interestingly, placentas derived from PE patients displayed a decreased level of HB-EGF (34). Based on our observation that gal-1 is responsible for the production of mature HB-EGF, we hypothesized that exogenous gal-1 is involved in activating HB-EGF on trophoblast cells. To test this hypothesis, we evaluated the level of sHB-EGF in trophoblasts upon gal-1 stimulation in vitro. As shown in Fig. 3D, exogenous gal-1 induced the maturation of HB-EGF in a dose-dependent manner. Interestingly, the level of sHB-EGF is significantly decreased in unstimulated trophoblast cells from fullKO mice compared with WT (Fig. S6D). Of note, stimulation of *Lgals1*-deficient trophoblasts with sHB-EGF resulted in similar B4GALNT2 expression levels than in *Lgals1* WT trophoblasts (Fig. 3E). Thus, exogenous gal-1 is necessary for the maturation of HB-EGF at the maternal–fetal interface; however, expression of B4GALNT2 can also be induced by sHB-EGF independently of the intracellular gal-1 expression.

## Discussion

**Fig. 4.**
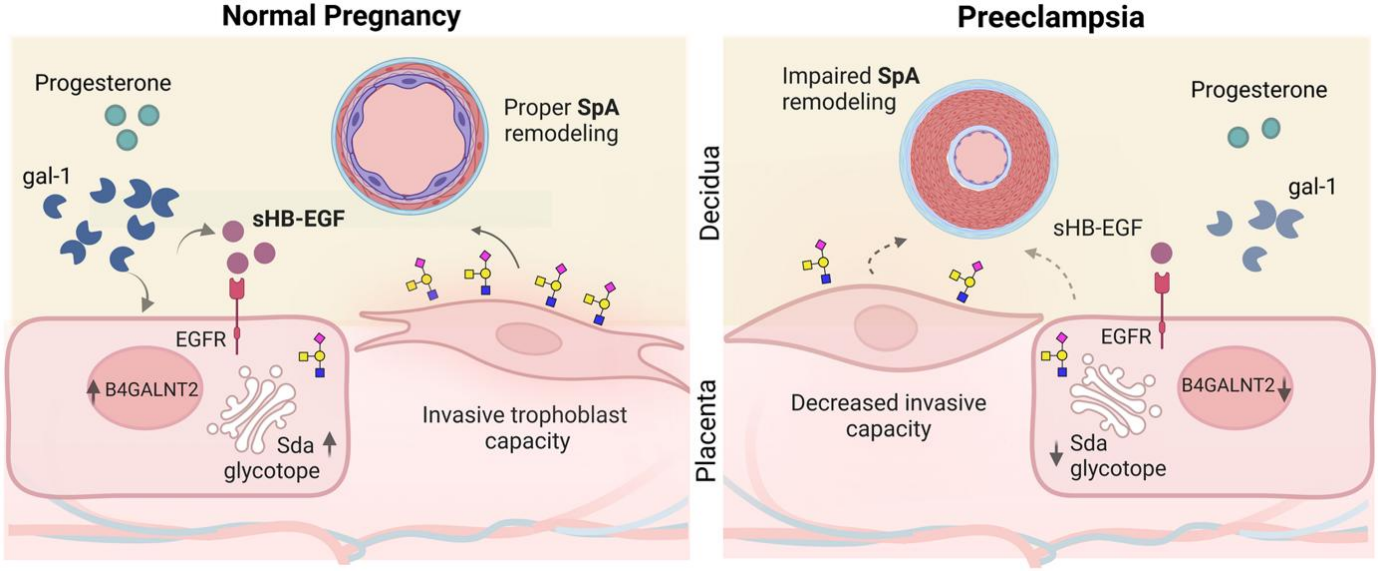
Schematic depiction of the maternal gal-1 effects on the regulation of trophoblast invasion and remodeling of the SpA. In normal pregnancy, maternal gal-1 induces sHB-EGF secretion, leading to B4GALNT2 activation and Sda-capped glycosylation. As a result, trophoblasts acquire an invasive phenotype necessary for SpA remodeling. In preeclampsia, reduced maternal gal-1 impairs sHB-EGF maturation by directly affecting B4GALNT2 activation and indirectly by altering the concentration of progesterone, a hormone that increases the availability of sHB-EGF. Inadequate trophoblast invasive capacity compromises the SpA remodeling and subsequent preeclampsia development.

For several decades, the contribution of the maternal niche to the etiology of PE has been unappreciated; however, recent studies utilizing in vitro approaches and global transcriptional profiling highlighted its critical importance. Nevertheless, the mechanisms by which the maternal niche impacts the placentation program are poorly understood. Galectins, particularly gal-1, contribute to maternal and placental niches’ healthy adaptation to pregnancy (11, 12). Interestingly, early studies noted that the lack of gal-1 in both compartments imposed a risk for PE development (17). In this study, we have used in vivo models to determine how maternal- and placental-derived gal-1 deficiency impacts pregnancy outcomes. We noted that gal-1 is more abundant in the maternal than the placental niche once pregnancy is established. The lack of gal-1 in the maternal compartment severely interferes with placenta efficiency, which results in an altered fetal maturity trajectory accompanied by a PE-like syndrome. In addition, our findings show that maternal-derived gal-1 regulates the invasive trophoblast capacity through the maturation of HB-EGF, which increases Sda terminal glycosylation (Fig. 4). Compensation by other galectin members (gal-3 and gal-9) is insufficient to overcome gal-1 absence in the maternal niche. Thus, we provide evidence of the unique function of the maternal compartment through this lectin in orchestrating a healthy pregnancy.

Particular attention has been paid over the last decades to the role of gal-1 at the maternal–fetal interface. In vitro studies carried out to understand the function of the placental-derived gal-1 (endogenous) showed that this lectin is required for the trophoblast invasive and immunoregulatory properties (e.g. HLA-G expression) necessary for optimal placentation (16). Furthermore, we previously showed a dramatic placenta insufficiency leading to FGR and PE development in fullKO (maternal/placental) dams (17). The results presented here using in vivo models demonstrate that loss of placental gal-1 (fplKO) has a modest impact on fetal maturity and placenta growth with no maternal manifestations of PE-like syndrome. Thus, inadequate placental-derived gal-1 is not sufficient to pose a threat to the pregnancy. On the other hand, this study revealed that gal-1 WT placentas were not fully functional when gal-1 was absent in the maternal niche, as mKO dams spontaneously developed late-gestational inadequate cardiovascular adaptation (clinical features of PE). Our findings provide evidence that maternal-derived gal-1 (exogenous) signaling is necessary to maintain a functional *Lgals1*-deficient placenta. Interestingly, we previously showed that dams in which gal-1 is blocked with Anginex, a specific gal-1 antagonist, are at risk of developing PE, mainly due to a dramatically abnormal trophoblast vascular remodeling profile (17). Indeed, our analysis of the placenta developmental stage on mKO dams showed a significant decrease in *Hand-1* and *Prl3d1* gene expression, which mainly characterize the Jz. This heterogeneous placenta layer plays a pivotal role in decidual invasion and SpA remodeling, among others. Notably, *Hand-1* mutant trophoblast cells are less invasive in vitro (35, 36), and *Prl3d1* is involved in maternal metabolism and maintains a constant supply of glucose and amino acids to the fetus (37). In line with these observations, mKO-derived placentas showed delayed trophoblast maturity involved in the transformation of uterine vasculature (SpA remodeling) which is associated with the development of PE (38). Uterine NK cells and invasive trophoblasts are critical for maternal vasculature remodeling during pregnancy (4). Therefore, we analyzed the impact of maternal-derived gal-1 deficiency on the tissue- and vascular-associated uNK cells. Decreased uNK cells associated with SpA walls in mKO dams agree with the exacerbated placental hypoxia. This is further amplified by the severe inflammatory placental phenotype in these mice, which manifests as inadequate maternal cardiovascular adaptation. Indeed, PE is associated with exacerbated immune activation caused by the imbalance of proinflammatory/regulatory/antiinflammatory cytokines which further promotes the endothelial dysfunction and subsequent clinical disease manifestation (39). These results provide further proof that a deficit of maternal-derived gal-1 is sufficient to cause PE disease.

During pregnancy, physiologic changes occur in the mother's circulation to meet the needs of the developing fetus. Early in pregnancy, an increase in the maternal blood volume and cardiac output without increasing the blood pressure is observed. This phenomenon is due to a general decrease in vascular resistance and is attributed to the remodeling of the spiral arteries (40). Remarkably when the vessel architecture of the uterus is developed, the increase in blood flow is compensated by sustained vasodilation of these existing vessels (41). Therefore, from mid-gestation, adequate uteroplacental blood flow depends on vasodilation and early-onset PE can be characterized by reduced capacity for vasodilation (1, 42). One of the principal vasodilators is NO, and its production is catalyzed by eNOS from L-Arg in the maternal endothelium (43). Moreover, NO is essential for the invasion of the CTBs into the maternal SpA and a reduction of NO biosynthesis or bioavailability has been associated with PE (44). Our results demonstrated a significant decrease in L-Arg and an increase in SDMA, an inhibitor of L-Arg transport (22) in mKO. The ratio L-Arg/ADMA, an index to measure eNOS activity (21), and the ratio L-Arg/SDMA are also down-regulated in mKO. Overall, these results showed a decreased production of NO in mKO. In these animals, placenta inflammation and an increased blood pressure were observed later in gestation, suggesting an impaired vascular adaptation due to the lack of maternal gal-1 is associated with PE-like syndrome. Like our results, a decrease of L-Arg levels and in the ratio L-Arg/ADMA was observed in patients with PE compared with controls (45, 46). We have previously demonstrated that in early gestation, the inhibition of gal-1-mediated angiogenesis by Anginex provokes PE-like symptoms (17). Normal pregnancy is associated with endothelium-dependent vascular adaptation, and especially gal-1 is a critical component of the vascular remodeling (47, 48). Our study design allowed us to uncover the role of gal-1 within the maternal vascular adaptations to pregnancy and its role in the Lab layer phenotype. Consistent with the maternal vascular remodeling defects in mKO mice, the fetal Lab showed increased branching morphogenesis and reduced lacunarity. It is important to note that Lab layer changes in the vascular compartment coincided with lower placental partial pressure of oxygen (PO_2_) in *Lgals1*-deficient mice (49), which directly links poor placental oxygenation and placental dysfunction and is consistent with the previously reported gal-1 multiple endothelial cell functions, including activation, proliferation, migration, tube formation, and sprouting at the maternal–fetal interface (50). Our results further highlight the importance of maternal-derived gal-1 in the development of PE, since the lack of gal-1 in this compartment, and not in the placenta, showed a severely impaired endothelial function.

The maternal niche, through several factors, tightly controls the invasive trophoblast capacity to enable proper placentation (51). Failure in trophoblast invasion and reduced SpA remodeling is associated with pregnancies complicated with PE (52). We have previously shown differential glycosylation of human trophoblast cell types, including the enrichment of polylactosamine-containing N-glycans that could act as receptors for galectins, on EVT involved in SpA remodeling (53). Notably, our present study revealed that mKO-derived placenta displayed altered glycosylation characterized by a reduction of complex N-glycans capped by the Sda epitope. The enzyme involved in Sda synthesis is B4GALNT2, previously known as β1,4GalNAcT-II (GALGT2) (54). This enzyme is up-regulated by progesterone in the mouse uterus and, during pregnancy, peaks at E10, coinciding with trophoblast invasion into the decidua (30, 55). Our findings of reduced levels of progesterone in fplKO and mKO dams compared with controls, combined with evidence of B4GALNT2 regulation by this hormone, highlight the significance of the cooperation between progesterone-gal-1 regulation pathways during placenta development; this synergy is also relevant in the immune adaptations required for a successful pregnancy (14, 15). However, whether the differential glycosylation (Sda-capped glycans) is involved in the invasive trophoblast capacity has not been determined. Our data demonstrate that the inhibition of B4GALNT2 reduced the invasion capacity of the murine SM9-1 and human HTR-8/SVneo trophoblast cell lines, suggesting a role of Sda terminal glycosylation on the invasive properties of trophoblast cells at the maternal–fetal interface. In the present study, we also showed that EVT *LGALS1* expression is dysregulated during PE as indicated by single-cell RNA-seq analysis, which, for the first time, associates the decidual gal-1 with the PE syndrome. We have previously suggested additional functions for the Sda epitope on (i) the murine zona pellucida 3 glycoprotein, which participates in sperm–egg binding (cell–cell interaction) (56), and (ii) bovine pregnancy-associated glycoproteins where the differential expression of Sda during pregnancy may influence the serum half-life of these proteins (57). Thus, the synthesis of the Sda-glycotope is likely regulated in a tissue-specific manner and could be under hormonal regulation. In this regard, it has been shown that HB-EGF regulates B4GALNT2 expression in mouse skeletal muscle (32). HB-EGF participates in pregnancy-associated processes, including embryo–uterine cross-talk during implantation in mice and humans (58, 59). Decreased placental HB-EGF was observed in pregnancies complicated with PE (34, 60). Here, we show that exogenous sHB-EGF induced the expression of B4GALNT2 in vitro of primary isolated trophoblast cells, indicating that HB-EGF activates the synthesis of the Sda-glycotope at the maternal–fetal interface. Interestingly, gal-1 induces HB-EGF expression and ectodomain cleavage increasing its bioavailability in the lung tumor microenvironment (61). We observed that exogenously added gal-1 induced the maturation of HB-EGF in a murine trophoblast cell line and B4GALNT2 expression. Thus, our results demonstrated that maternal-derived gal-1 activates the trophoblast proinvasive program through HB-EGF maturation and differential Sda terminal glycosylation. This molecular pathway may be involved in one of the several identified gal-1 protective functions during pregnancy.

In summary, we demonstrated that the maternal niche through gal-1 expression modulates the placental functional development and that this lectin promotes maternal cardiovascular adaptation during pregnancy. Furthermore, our findings suggest that designing therapeutic strategies to improve maternal gal-1 expression could improve pregnancy outcomes and reduce the increased cardiovascular risk post-preeclampsia.

## Materials and methods

### Mice and tissue collection

*Lgals1* ^+/+^ and *Lgals1*^−/−^ mice (129/P3J background) were acquired and maintained, and in vitro fertilization timed pregnancies were established as described (19). *Lgals1^+/+^* embryos were transferred to *Lgals1^−/−^* dams to create the mKO, whereas a fplKO was obtained by transferring *Lgals1^−/−^* embryos to *Lgals1^+/+^* recipient dams. Animal care and all experimental procedures were performed according to Charité/University Medical Center Hamburg-Eppendorf institutional guidelines and conform to requirements of the German Animal Welfare Act (Berlin/Hamburg). Timed pregnant mice were evaluated at embryonic day (E) 7, 13, and E17 (*n* = 6–8 mice per group). Offsprings from the different experimental models were evaluated on PN day 28. Whole implantations were frozen for cryostat sections or formalin fixed for paraffin sections on E7 and E13. Placental and decidual tissues were separated on E13 and frozen for isolation of total protein or total RNA according to standard procedures (15). Placental weight was recorded in late gestation (E17). Fetal development was analyzed by Theiler stage and body weight as described previously (62). Details are available in Materials and Methods S1.

### ELISAs

Quantification of murine gal-1 and sHB-EGF were performed using the mouse gal-1 DuoSet enzyme-linked immunosorbent assay (ELISA) (R&D Systems; DY1245) and mouse HB-EGF DuoSet ELISA (R&D Systems; DY8239) respectively, following the manufacturer's recommendations. Details are available in Materials and Methods S1.

### Cardiovascular adaptation analysis in pregnant mice

Systolic and diastolic pressure was measured in the tail artery in pregnant females from E8 to E17 with a computerized, noninvasive tail-cuff acquisition system (CODA System; Kent Scientific) as described previously (16). In addition, L-Arg, ADMA, and SDMA were quantified from blood samples stored at −80°C applying a validated tandem mass spectrometric assay (MS/MS) (63). Details are available in Materials and Methods S1.

### N-Glycan profiling of placental samples

N-glycan analysis from mouse placenta was carried out by MALDI-TOF/TOF according to the previous study (64). Details are available in Materials and Methods S1.

### Cell culture and in vitro treatments

B4GALNT2 expression was inhibited in the mouse SM9-1 and human HTR-8/SVneo trophoblast cell lines by siRNA (Origene, SR416377 and SR314851, respectively) according to the manufacturer's instructions. After 36 h, cells were harvested to analyze *B4GALNT2* mRNA or protein expression or to perform the invasion assay. For gal-1 treatments, 6-h FBS starvation was previously performed in SM9-1 cells and then incubated with 10 or 100 ng/ml recombinant gal-1 (Peprotech, 450–39). After 24 h, supernatant was collected, and cells were harvested to protein isolation and western blot analysis. Trophoblast cells were isolated from E13 placenta as previously described (65). A 6-h serum starvation was performed, and then trophoblasts were treated with 0.1, 1, or 10 nM of recombinant HB-EGF (R&D, 259-HE/CF). After 24 h, cells were collected and cytospin was performed to evaluate B4GALNT2 expression. Details are available in Materials and Methods S1.

### Statistics

Numerical data were analyzed with D’Agostino–Pearson normality test followed by ANOVA and Tukey's test or Kruskal–Wallis analysis and Dunn's test. Categorical variables are presented as frequency and assessed by chi-square test with post hoc Bonferroni adjustment. Statistical significance was designated as *P* < 0.05 analyzed by GraphPad Prism 9.0.

## Supplementary Material

pgad247_Supplementary_Data

## Data Availability

All the codes to perform and reproduce the single-cell analysis presented in this manuscript are available at https://github.com/Manu-1512/Galectin-in-placenta-development-and-preeclampsia.
