# Supplementary material for: Maternal-derived galectin-1 shapes the placenta niche through Sda terminal glycosylation: Implication for preeclampsia
Source: PNAS Nexus. 2023 Aug 1;2(8):pgad247. doi: 10.1093/pnasnexus/pgad247 (PMC10416815; doi:10.1093/pnasnexus/pgad247)
Supplement: pgad247_Supplementary_Data [file pgad247_supplementary_data.docx]

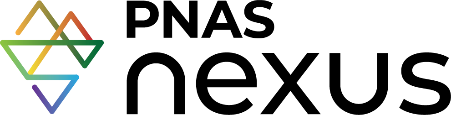


**Supporting Information for**

Maternal-derived galectin-1 shapes the placenta niche through Sda terminal glycosylation: Implication for Preeclampsia

Yiran Xie, Fangqi Zhao, Nancy Freitag, Sophia Borowski, Yiru Wang, Charlotte Harms, Poh-Choo Pang, Juliette Desforges, Tianyu Wen, Edzard Schwedhelm, Manvendra Singh, Ralf Dechend, Anne Dell, Stuart M. Haslam, Gabriela Dveksler, Mariana G. Garcia, Sandra M. Blois

Email: [s.blois@uke.de](mailto:s.blois@uke.de)

**This PDF file includes:**

Supporting Materials and Methods

Figures S1 to S6

Tables S1 and S2

SI References

Supporting Information

**SI Material and Methods**

***In vitro* fertilization (IVF).** *Lgals1^-/-^* and *Lgals^+/+^* females (8-12 weeks old) were induced to superovulation by intraperitoneal (i.p.) injection of Pregnant Mare's Serum Gonadotropin (PMSG, Pregmagon, Covetrus DE GmbH, 5 I.U. per 100 µL PBS). Forty-eight hours later, they were i.p. injected with human chorionic gonadotropin (hCG, Ovogest, Intervet Deutschland GmbH, 2.5 I.U. per 100 µL PBS) and oocyte collection was performed after 15 hours. For the collection of spermatozoa, male mice (3-6 months old) were sacrificed, and the cauda epididymidis removed. The cauda epididymidis was placed on a petri dish with mineral-oil covered with CARD FERTIUP Preincubation Medium (Cosmo Bio Co. LTD.) that was pre-incubated overnight at 37°C in the presence of 5% CO_2_. A cut in the cauda epididymidis with a 26-gauge needle was performed and the clots of spermatozoa released from the cauda epididymidis were introduced into the drop of Preincubation Medium. The remaining tissue from the mineral oil was removed and the sperm was allowed to capacitate for 40-60 min in incubator (37°C, 5% CO_2_). For oocyte collection, the superovulated female mice were sacrificed by cervicale dislocation. After exposure of the uterine horns, oviducts and ovaries, the uterus was grasped with forceps and cut it close to the oviduct. A cut above the ovary was performed and the tissue was transferred into oil phase of the 35 mm dish with the oil covered CARD MEDIUM (Cosmo Bio Co. LTD.). The ampulla, which is the swollen part of the oviduct, were the cumulus-oocyte-complexes are visible, was opened and the cumulus-oocyte-complexes were release into the drop of CARD MEDIUM. For IVF, 2-5 µL of the sperm suspension were added to the drop of CARD MEDIUM containing the cumulus-oocyte-complexes and were incubated for 4-6 hours in an incubator (37°C, 5% CO_2_). After an incubation of 4-6 hours, the cells (zygotes) were washed two times in separate drops of pre-incubated CARD MEDIUM. For washing the embryos, they were drawn into a pipette with clean medium and released into a clean drop, repeating this procedure twice under the microscope to remove aberrant embryos and cumulus cells. The cells were incubated overnight in an incubator (37°C, 5% CO_2_) and the 2-cell stage embryos were counted the next morning.

Pseudo-pregnant foster mice (mated with vasectomized males) at gestation day 0.5 were weighed and anesthetized (i.p. injection of 100 µL per 10 g of body weight of 122 mg/kg Ketamine and 10 mg/kg Xylazine). Analgesia was administered (100 µL of analgesia per 10 g body weight) under the skin in the neck when the anesthesia is effective (Rimadyl: 10 mg/kg). After 70% ethanol disinfection, a small incision in the skin above the spinal column just below the rib cage was performed. A 5 mm opening allowed to locate the ovary (orange) or fat pad (white), a serrefine clamp was attached to the fat pad near the ovary and relocated the distal part of the uterus, oviduct and ovary onto the back of the mouse and the bursa over the infundibulum was opened with micro-spring scissors. Air and medium were aspirated in alternate intervals of 2-3 mm into the glass capillary and six embryos were draw into it. The tip of the capillary was inserted into the infundibulum and gently push towards the ampulla. Six embryos and 2-3 air bubbles were transferred to each oviduct, the capillary was removed, and the fat pad released. After the relocation of the uterus, oviduct and ovary into the body cavity, the muscle was closed with a single knot suture and the fur with wound clips. The mouse was let for recover from the anesthesia on a hot plate.

**Tissue homogenates.** Tissue lysates of mouse placenta or decidua on E13 were prepared by mechanical homogenization in phosphate-buffered saline (PBS) with metal lysis beads with shaking at 20 Hz for 10 min (TissueLyser II, Qiagen). Samples were then centrifuged at 13,000 x g at 4°C for 10 min, the supernatant was collected and stored at -80°C. Protein concentrations were determined by Bradford assay. Tissue lysates derived from placenta and decidua were used to determine the gal-1 levels by ELISA and Luminex was performed in placenta lysates to characterize the cytokine milieu.

**Murine galectin-1 and mouse HB-EGF ELISA.** A 96 well half-area plate (Corning; CLS3690) was coated with the capture antibody overnight. After washing the plate, blocking was performed by adding 1% bovine serum albumin (BSA) in PBS. Plates were washed and a standard curve was prepared in a two- fold dilution series (from 8,000 to 125 pg/mL gal-1; from 2,000 to 31.25 pg/mL HB-EGF), and mouse placenta or decidua lysates (for gal-1 measurement) or SM9-1 cell supernatant (for HB-EGF measurement) were applied and incubated 2 hours. After washing, the detection antibody was incubated 2 hours followed by streptavidin-HRP for 20 min. After washing, a 3,3’,5,5’-tetramethylbenzidine (TMB) substrate solution was added and incubated for 5 min (gal-1) or 20 min (HB-EGF) in the dark. The colorimetric reaction was stopped with 4N H_2_SO_4_ and the optical density (OD) was determined at 450 nm. All incubations were performed at room temperature (RT). The gal-1 and HB-EGF levels were calculated from the standard curves with a four parameter logistic (4-PL) curve fit. The obtained concentration was normalized by the amount of protein in each sample.

**Blood pressure evaluation**. For blood pressure measurements, mice were placed in plastic holders and the body temperature was maintained between 34°C and 36°C with infrared heating. The CODA system uses volume-pressure recording technology to detect changes in tail volume that correspond to systolic and diastolic pressures (in millimeters of mercury).

**L-arginine and dimethylarginine measurement.** L-arginine (L-Arg), asymmetric dimethylarginine (ADMA) and symmetric dimethylarginine (SDMA) were quantified by tandem-mass spectrometric assay. In brief, 25 µL of serum were diluted with stable isotope labelled internal standards solved in 100 µL methanol. After protein precipitation and evaporation of supernatants to dryness, analytes were converted to their butyl ester derivatives and subjected to ultra-performance liquid chromatography (UPLC) coupled to positive electrospray ionisation (ESI+) MS/MS. L-arginine metabolite concentrations were calculated by division of peak area ratios of metabolites over internal standards.

**Albumin-to-Creatinine Ratio.** Twenty-four–hour urine samples were collected, cleared by centrifugation, and albumin and creatinine levels on E17 were determined (Exocell; 1011 and 1012, respectively) according to the manufacturer’s instructions. Briefly, for albumin measurement, standards were prepared in a two-fold dilution series (from 10 to 0.156 μg/mL). Standards and samples were added to the supplied test microtiter plates. The anti-albumin antibody was applied and incubated for 30 min at RT. After washing, HRP-conjugated antibody was added and incubated for 30 min at RT. The plate was washed and incubated for 10 min with color developer and after that, the OD at 450 nm was determined. The albumin concentrations were calculated from the standard curve. For creatinine determination, the samples (diluted 1:5) and the ready-to-use standards were added to a 96 well half-area plate (Corning; CLS3690) following with the incubation of picrate working solution. After 10 min, the OD at 500 nm was determined (= OD picrate). The acid reagent was added and incubated for 5 min and the OD was measured at 500 nm (OD picrate + acid). The differences of OD picrate and OD picrate + acid were calculated for every sample and the creatinine levels were calculated from the standard curve. For calculating the albumin-to-creatinine ratio (ACR) expressing the amount of albumin (in µg) per milligram of creatinine, the albumin concentrations were divided by the creatinine levels for every sample.

**Histology analysis.** To quantify the wall thickness of mesometrial arteries, the ratio of the total vessel to the lumen area was calculated on H&E staining as described previously (9). The quantification of tissue-associated (ta) and vascular-associated (va) uNK were performed by the Dolichos biflorus agglutinin (DBA)/ Periodic Acid Schiff’s (PAS) staining as previously described (10). Uterine NK (uNK) cells were analysed by DBA^+^PAS^+^ staining since DBA-lectin reactivity defines NK cells that have homed to mouse decidua and mouse uNK are identified as lymphocytes containing PAS reactive cytoplasmic granules. PAS staining was used to determine the glycogen content stored by glycogen cells in the junctional zone of E13 placentas. The fractional area of PAS-positive glycogen trophoblast cells was analyzed by digitally selecting the Lab and using the color deconvolution tool for hematoxylin and PAS staining on ImageJ. Areas with only PAS staining were extracted and by adjusting the threshold with ImageJ, the whole Lab [1] and stained PAS^+^ cells [2] were selected and calculated as [2]/[1]. Analysis of vascularization was performed on Isolectin B4 stained sections using Angiotool (11), which allows automatic identification of vascular networks using multiscale Hessian analysis and skeletonization.

**Endoglin, Dolichos biflourus (DBA)/perforin, CD31/cytokeratin, and alpha-Smooth Muscle Actin (α-SMA) immunofluorescence.** Cryosections from whole implantations on E7 (for endoglin and DBA/perforin analysis) or E13 (for CD31/ cytokeratin and α-SMA evaluation) were cut at 8 μm, washed in Tris-buffered saline (TBS) and blocked with 2% goat normal serum (for endoglin, CD31/cytokeratin and α-SMA) or Proteinblock (Dako #X0909, for DBA/perforin) and incubated overnight at 4°C with the primary antibody against endoglin (1:200, Santa Cruz Biotechnology, sc-18893) or biotin-conjugated Dolichos biflourus (DBA) lectin (1:2000, Sigma, #L6533-5mg/mL) plus antibody against perforin (1:50, Santa Cruz Biotechnology, sc-9105) or biotin-conjugated antibody against CD31 (1:200, BioRad, MCA2388BT) plus antibody against cytokeratin (1:500, Dako, Z0622) or antibody against alpha-smooth muscle action (α-SMA) (1:200, Cell Signaling Technologies, 19245). Negative controls were included, in which primary antibodies were replaced by irrelevant IgG. After washing, sections were incubated 1 hour at RT with secondary antibodies (TRITC-conjugated 1:200, Jackson ImmunoResearch, 112–025-167 for endoglin staining; AlexaFluor488 goat anti-rabbit 1:200, Jackson ImmunoResearch, 111-545-003 for perforin, cytokeratin and α-SMA staining; streptavidin-Tetramethylrhodamine 1:500, Invitrogen, S-870 for DBA and CD31 staining) for 1 hour at RT in a humid chamber. After washing, nuclei were counterstained by incubating 5 min in DAPI solution, followed by washing and mounting in Prolong Gold (P36930; Invitrogen). Sections were digitally scanned by a high-resolution fluorescence slide scanner (Pannoramic MIDI BF/FL, 3DHISTECH Ltd.) and the analysis of the images was performed using CaseViewer 2.4 software (3DHistech Ltd., Budapest, Hungary). Endoglin staining was analyzed in the vascular zone (VZ) by Angiotool as previously described (11). The percentage of degranulated uNK cells was calculated by dividing the number of uNK cells (DBA^+^ cells) surrounded by perforin staining that did not overlap with the DBA staining (designated as degranulated uNK cells) by the total number of uNK cells. For the quantification or the % of α-SMA^+^ vessels in the decidua basalis, vessels are considered positive if they have over 40% α-SMA^+^ staining.

**Luminex analysis.** Lysates from E13 placenta were analyzed for a panel of 17 cytokines (GM-CSF, IFN-γ, TNF-α, IL-1ß, IL-2, IL-4, IL-5, IL-6, IL-9, IL-10, IL-12p70, IL-13, IL-17A, IL-18, IL-22, IL-23 and IL-27) by Luminex bead-based multiplex assay using a mouse Th1/Th2/Th9/Th17/Th22/Treg Cytokine 17-Plex ProcartaPlex Panel (Invitrogen, EPX170-26087-901) according to the manufacturer’s instructions. For undetectable cytokines (below the lowest detection limit), we assigned a value of 0.1 pg/mL for analysis.

**Galectin analysis (iPATH Multiplex).** Paraffin sections were dewaxed and subjected to a heat-induced epitope retrieval step. Endogenous peroxidase was blocked by hydrogen peroxide prior to incubation with anti-CD31 (clone D8V9E, Cell Signaling Technologies) followed by incubation with EnVision+ HRP-labelled polymer (Agilent). For visualization, the OPAL system was used according to manufacturer´s instructions (Akoya Biosciences). Proteins were then inactivated and sections incubated with anti-gal-1 (polyclonal Ab #GTX 101566, GeneTex) followed by incubation with the EnVision+ polymer (Agilent) and the OPAL system (Akoya Biosciences). This staining cycle was repeated with anti-gal-3 (polyclonal Ab #GTX 125897, Gene Tex) and anti-gal-9 (polyclonal Ab, #ab69630, Abcam). Nuclei were stained using DAPI and slides were coverslipped in Fluoromount G (Southern Biotech). Multispectral images were acquired using a Vectra® 3 imaging system (Akoya Biosciences). Pseudocolours are shown with gal-1 in green, gal-3 in red, gal-9 in blue, CD31 in cyan and nuclei in white.

**N-glycan profiling of placental samples.** Tissues were sonicated and homogenized before reduction and carboxymethylation. Trypsin digestion was performed to cleave the glycoprotein into peptides and glycopeptides. N-glycans were subsequently released from glycopeptides with Peptide N-Glycosidase F digestion. Samples were purified, lyophilized, permethylated and dissolved in methanol before spotting onto the metal plates used for analysis on an AB Sciex 4800 MALDI-TOF/TOF mass spectrometer. The MALDI matrix was DABP. Mass spectra were visualized with Data Explorer 4.9 Software (AB Sciex) and glycan structures were built manually with the aid of GlycoWorkBench (12). The proposed structures were based on monosaccharide compositions derived from the molecular ion m/z values, knowledge of N-glycan biosynthetic pathways, and MS/MS derived fragmentation.

**Analysis of scRNA-seq datasets.** Firstly, we downloaded the processed matrix from E-MTAB-6701, PMID:30429548. The annotations were transferred from each cell specifications provided by the original publications. We then used Seurat (v3.1.1) within the R environment (v3.6.0) for filtering, normalization and cell-type identification for the given samples. The following data processing was done: (1) Filtering. We kept the cells with minimum and maximum of 1,000 and 5,000 genes expressed (≥1 count), respectively. Moreover, cells with more than 5% of counts on mitochondrial genes were filtered out. After filtering, there were 64,782 cells. (2) Data normalization. Gene UMI counts for each cell were divided by the total number of counts in that cell and multiplied by 10,000. These values were then natural-log transformed. (3) Cell-type identification. Integration of scRNA-seq data with all samples was performed using top 2000 variable features. Clustering was performed using “FindClusters” function with default parameters except resolution was set to 0.1 and first 20 PCA dimensions were used in the construction of the shared-nearest neighbor (SNN) graph and to generate 2-dimensional embeddings for data visualization using UMAP. Cell types were assigned based on the annotations provided by the original publication. The similar approach was used to analyze the data from healthy and preeclampsia samples. Here, CTB, EVT and STB was identified using their marker genes as a bait. We used "HLA-G","FN1" genes for EVT, "CYP19A1", "CGA" genes for STB and "PAGE4","PEG10" genes for identifying CTB lineages from the data.

We identified the major clusters corresponding to CTB, STB, EVTB, Macrophages and stromal cells. We defined the cell type for each cluster according to the known markers. We then used Monocle2 (https://github.com/cole-trapnell-lab/monocle-release) single-cell trajectory analysis and cell ordering along an artificial temporal continuum using the top 500 differentially expressed genes between CTB, STB and EVT cell populations. The transcriptome from each single cell represents a pseudotime point along an artificial time vector that denotes the progression of CTB to STB and EVT. Finally, we plotted the trajectory, pseudotime and *LGALS1* gene expression using R codes. All the codes to perform and reproduce the single cell analysis presented in this manuscript is available at <https://github.com/Manu-1512/Galectin-in-placenta-development-and-preeclampsia>.

**Cell line culture, trophoblast isolation and *in vitro* treatments.** The mouse trophoblast cell line SM9-1 was cultured in DMEM/F12 (Gibco, 11320-074) supplemented with 10% fetal bovine serum (FBS, Gibco, 10082147) and the human trophoblast cell line HTR-8/SVneo in RPMI1640 (Gibco, 21875-034) supplemented with 5% FBS, and maintained in a 37°C humidified incubator with 5% CO_2_. To inhibit B4GALNT2 expression 10 nM of a specific siRNA or a scrambled (Scr) sequence (Origene, SR416377 for mouse and SR314851 for human B4GALNT2) was delivered into the cells using SiTran 2.0 reagent (Origene, [TT320001](https://www.origene.com/catalog/others/transfection-reagents/tt320001/sitran-20-sirna-transfection-reagent-05-ml)) according to the manufacturer’s protocols. The isolation of trophoblast cells from E13 placenta was carried out by enzymatically digestion of the fresh tissue for 20 min at 37°C with 0.1% collagenase (Sigma-Aldrich, C2139) and 0.0025 % DNAse I (Sigma-Aldrich, D4263). After incubation, the enzyme activity was stopped with FBS, and the cells resuspended in RPMI1640. Cell suspension was filtered through a 50-mm mesh, laid onto a Percoll gradient (70-75% diluted with HBSS) and centrifuged for 25 min at 1,200 x g. The 30 - 45% Percoll layers with the trophoblasts were collected, the cells washed and cultured in RPMI1640 - 10% FBS for 24 hours. Cytospins were prepared using cytocentrifuge (Shandon Cytospin 2, Thermo Fisher Scientific). Briefly, trophoblast cells were resuspended at a density of 5x10^5^ cells/ml and 50 μL suspension loaded into disposable sample chambers (Single Cytofunnel, Thermo Fisher Scientific, 5991040). After centrifugation at 800 rpm for 5 min, slides were allowed to air dry for 30 min and then fixed with cold acetone for 10 min.

**B4GALNT2 expression analysis.** The expression of B4GALNT2 was evaluated in E13 implantation sites and in isolated trophoblast cells from E13 placenta (cytospin). The slides were incubated overnight at 4°C with anti-B4GALNT2 (USBiological Life Sciences, 362247) and subsequently with HRP-conjugated secondary antibody (Jackson Immuno-Research, 111-035-047) for 1 hour at RT. The signal was detected by incubating at RT with diaminobenzidine-peroxidase substrate solution. Cytospins were digitally scanned by Axio Scan.Z1 slide scanner (Carl Zeiss, Inc.) and the staining intensity was scored by a subjective scale (0, negative; 1, weak; 2, moderate; 3, strong) on virtual scans using ZEN lite 3.4 (Zeiss) by two independent examiners blinded to group allocation. The immunohistochemical results were evaluated by a semiquantitative approach using the H-score as previously described (13).

**Invasion Assays.** Cell invasion was evaluated using a Transwell invasion assay. Cell culture inserts (8 µm, Sarstedt, 83.3932.800) were coated with Geltrex (Gibco, A14132-02) diluted 1/10 with culture media. SM9-1 or HTR-8/SVneo cells (1x10^5^) were seeded in the inserts and serum containing media (20% FBS) was added into the lower wells and plates were incubated at 37°C overnight or 4 hours respectively. Non-invaded cells from the upper surface of the insert were removed by scrubbing with cotton swabs and the cells on the under surface were fixed with 2% formaldehyde and the nucleus stained with DAPI. For each condition, 10x magnification pictures were taken and cells were manually counted; the experiment was repeated three times.

**RNA isolation and quantitative real-time PCR (qPCR).** Total RNA was isolated from E7 implantation sites, E13 placenta. SM9-1 or HTR-8/SV neo cells with the RNeasy Plus Universal Mini Kit (Qiagen, 73404). cDNA was generated with random primers (Invitrogen) from 1 μg RNA in 20 μL with Superscript II (Invitrogen, 18064022) and quantitative real-time PCR was performed using the QuantStudio5 system (Applied Biosystems). Each reaction had a total volume of 10 μL containing 2 μL cDNA, 5 μL Power SYBR Green PCR master mix (Applied Biosystems; 4367659), 3 μL Diethylpyrocarbonate (DEPC) water and 500 nM of the appropriate forward and reverse primers. Primer sequences are given in Table S1 (mouse) and S2 (human). The following PCR program was used: 2 min at 50°C, 10 min at 95°C, 40 cycles of 15 sec at 95°C, and 60 sec at 60°C. Then, a melting curve analysis was performed 15 sec at 95°C, 1 min at 60°C and 15 sec at 95°C) to verify amplification specificity. The relative expression was calculated as 2^−dCt^, in which Ct= Ct gene of interest - Ct reference gene. For decidualization and differentiation genes, a Z-score normalization was performed and data was presented as heatmap.

**B4GALNT2 Western blot.** Protein extracts from SM9-1 or HTR-8/SVneo cells were obtained by mechanical dissociation in RIPA buffer with protein inhibitor. Protein concentrations were determined by Bradford Assay (BioRad). Twenty micrograms of total protein were then separated by NuPAGE 4-12% Bis-Tris gel (Thermo Fisher Scientific) and transferred onto Hybond-P PVDF membranes (Amersham Biosciences). After blocking, the blots were incubated overnight at 4°C with anti-B4GALNT2 (1:500; Bio-Techne #NBP1-91229) and HRP-conjugated antibody (Jackson Immunoresearch #111-035-047) or ß-actin HRP-conjugated (1/75000; Sigma #A3854). Signals were developed using SuperSignal West Femto (Thermo Scientific #34094) and visualized with Amersham Imager 600 (GE). Quantifications were performed by using ImageJ software. B4GALNT2 signals were normalized to ß-actin and control condition was arbitrarily set to 1.

**Progesterone.** Briefly, steroids were extracted from the plasma using diethylether and reconstituted in steroid-free human serum, according to the manufacturer’s protocol (DRG Instruments). The extracted samples were assayed in microtiter wells coated with progesterone-specific serum and incubated with horseradish peroxidase-conjugated progesterone for 60 min at room temperature. After washing, substrate solution (high sensitive tetramethylbenzidin) was added and incubated for 15 min before adding stopping solution (0.5 M H_2_SO_4_). Progesterone concentration was calculated based on a simultaneously run standard curve. Sensitivity of the assay was 0.05 ng/mL. Intra-assay coefficient of variation: 5.2–8.3%; inter-assay coefficient of variation: 6.5– 9.9%.


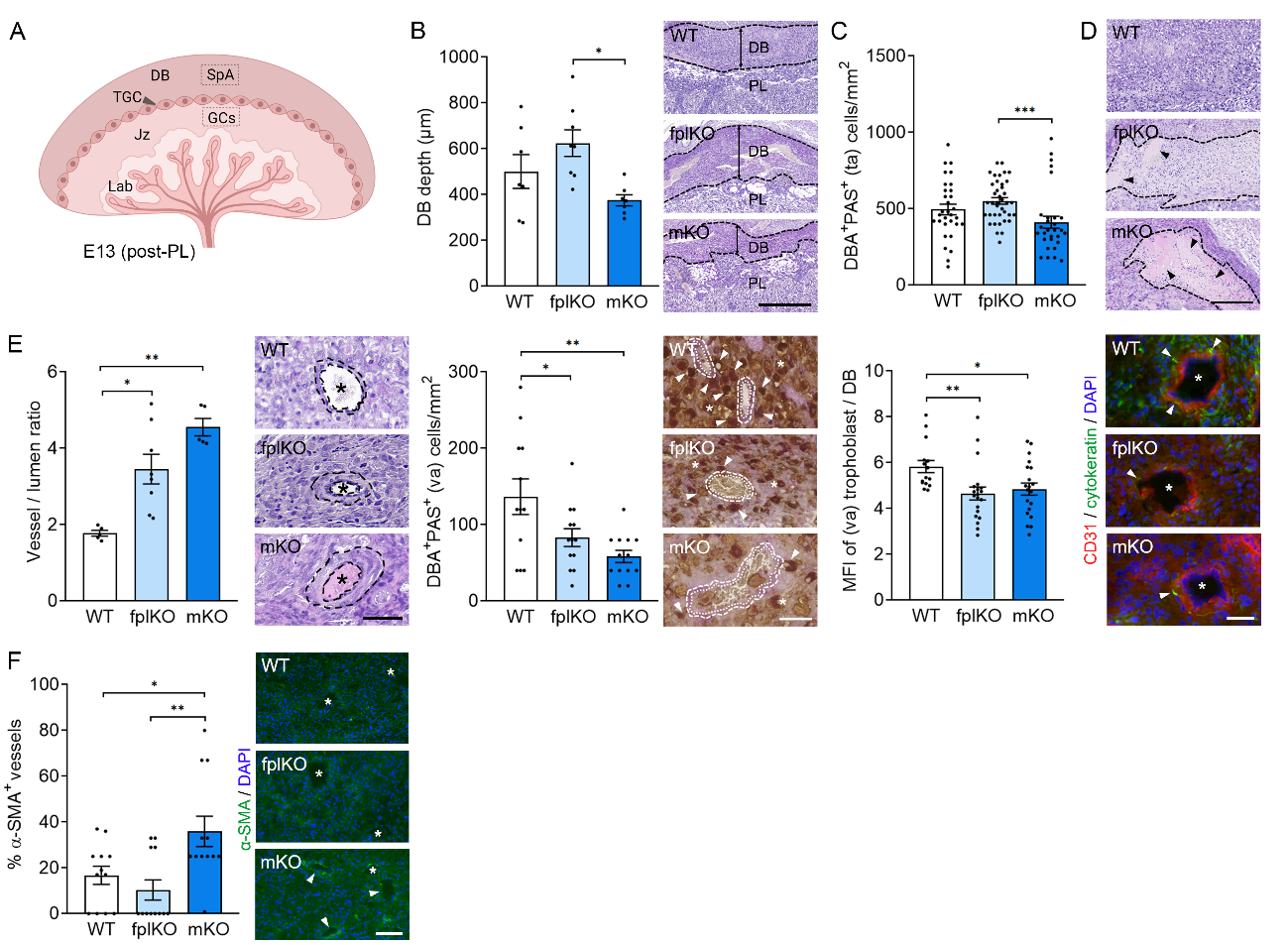


**Fig. S1.** Maternal gal-1 deficiency impacts on placentation. A) Schematic representation of placenta on E13. During the post-placentation (post-PL) period, spiral artery (SpA) remodelling is analyzed in decidua basalis (DB). The placenta consists of a junctional zone (Jz) where the glycogen cells (GCs) are located, and the labyrinth (Lab) layer. The placenta is delineated from the maternal decidua by a trophoblast giant cell (TGC) layer. B) Decidua basalis (DB) depth was calculated on E13 in *Lgals1*^+/+^ wild type (WT), fetal-placental knockout (fplKO) and maternal knockout (mKO) dams on E13 (n=7/ group). Representative images of H&E staining (right) showing the DB (dotted lines) and the placenta (PL) layers. Scale bar: 500 μm. C) Number of mature tissue-associated (ta) uNK cells (DBA^+^ PAS^+^) were counted in 5 to 6 regions in the decidua basalis and normalized to the area of the region (n = 7/ group). D) Representative H&E stained decidua basalis on E13. Dashed lines delineate the site of inflammation and necrosis areas are marked with arrows. Scale bar: 200 μm. E) *Left* Spiral artery (SpA) remodelling was evaluated by the wall thickness (vessel / lumen ratio) on H&E staining in the decidua basalis. Representatives examples of H&E staining showing in the SpA (asterisks), the inner and outer wall of the vessels (dotted lines). Scale bar: 50 µm. *Middle* Number of mature vascular -associated (va) uNK cells (DBA^+^ PAS^+^) were evaluated in in 5 to 6 regions within the decidua basalis and normalized to the area of the region (n = 7/ group). Representative images of the SpA (dotted line) and the presence of va-uNK cells (arrows) and ta-uNK cells (asterisks). Scale bar: 50 μm. *Right* Plot showing the mean fluorescent intensity (MFI) analysis of vascular associated trophoblasts within the SpA as evaluated by immunofluorescent staining for CD31 (vascular marker, red) and cytokeratin (trophoblast cells, green) 5 regions within the decidua basalis (n = 5/ group). Representative images depicting the SpA (asterisks) and the vascular associated trophoblast cells (arrows). Scale bar: 50 μm. F) Quantification of % α-SMA^+^ vessels. Representative pictures of SpA (asterisks) in the decidua basalis stained with α-SMA (arrows). Scale bar: 100 µm. In all figures, data are presented as the mean ± SEM. In Fig. S1B and S1E *middle* **P*<0.05 and ***P*<0.01 using one-way ANOVA followed by Tukey’s test and in Fig. S1C*,* S1E *left-right* and S1F **P<*0.05*, **P<*0.01 and ****P*<0.001 using Kruskal-Wallis followed by Dunn’s test. WT, *Lgals1*^+/+^; fplKO, gal-1 fetal-placental deficiency mice; mKO, gal-1 maternal deficiency mice.


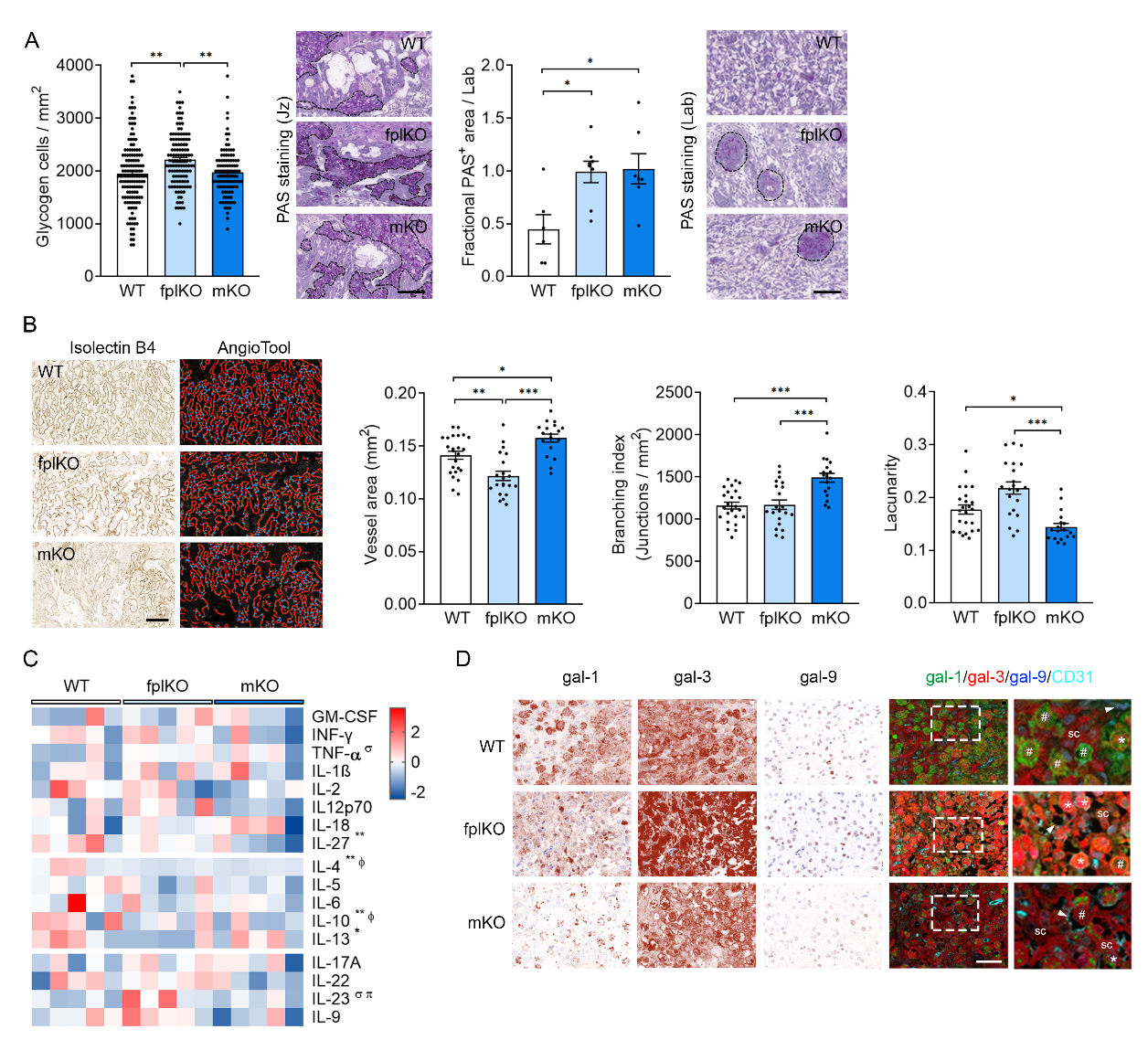


Fig. S2. Maternal gal-1 deficiency influences placenta well-being. A) *Left* Glycogen cells (GC) in the junctional zone (Jz) of the placenta were stained with periodic acid Schiff (PAS) and counted in 15 regions within the Jz on E13 and normalized to the area of the region (*n* = 6/ group). *Right* Fractional area of PAS-positive glycogen cells in the labyrinth (Lab) were evaluated on E13. B) Representative images of Isolectin B4 stained sections (*left panel*) for visualization of the labyrinth vascular networks. Inverted example images of the vascular network analyzed with AngioTool (*right panel*), depicting the skeletonized vessel network in red and branching points (vessel junctions) in blue. Scale bar: 100 µm. Quantitative results of AngioTool analysis of the vessel area, branching index and lacunarity on E13 in 5 regions within the Lab and normalized to the area of the region (n=6/ group). C) Heatmap showing the expression of cytokines analyzed by Luminex on placenta tissue homogenates. Data were previously normalized by Z-score (n=5/ group). D) Expression of gal-1, gal-3 and gal-9 in the decidua basalis using iPATH Multiplex. Scale bar: 50 µm. Higher magnification (right panel) to show decidual stromal cells (sc), natural killer cells (#), invasive trophoblast cells (asterisk) and endothelial cells (arrow). In all figures, data are plotted as mean ± SEM. In Fig. S2A and S2B **P*<0.05, ***P*<0.01 and ****P*<0.001 using one-way ANOVA followed by Tukey’s test and in Fig. S2C **P*<0.05 and ***P*<0.01 mKO vs WT; ^σ^*P*<0.05 and ^φ^*P*<0.01 fplKO vs WT and ^¶^*P*<0.05 fplKO vs mKO using one-way ANOVA followed by Tukey’s test*.* WT, *Lgals1^+/+^*; fplKO, gal-1 fetal-placental deficiency mice; mKO, gal-1 maternal deficiency mice.


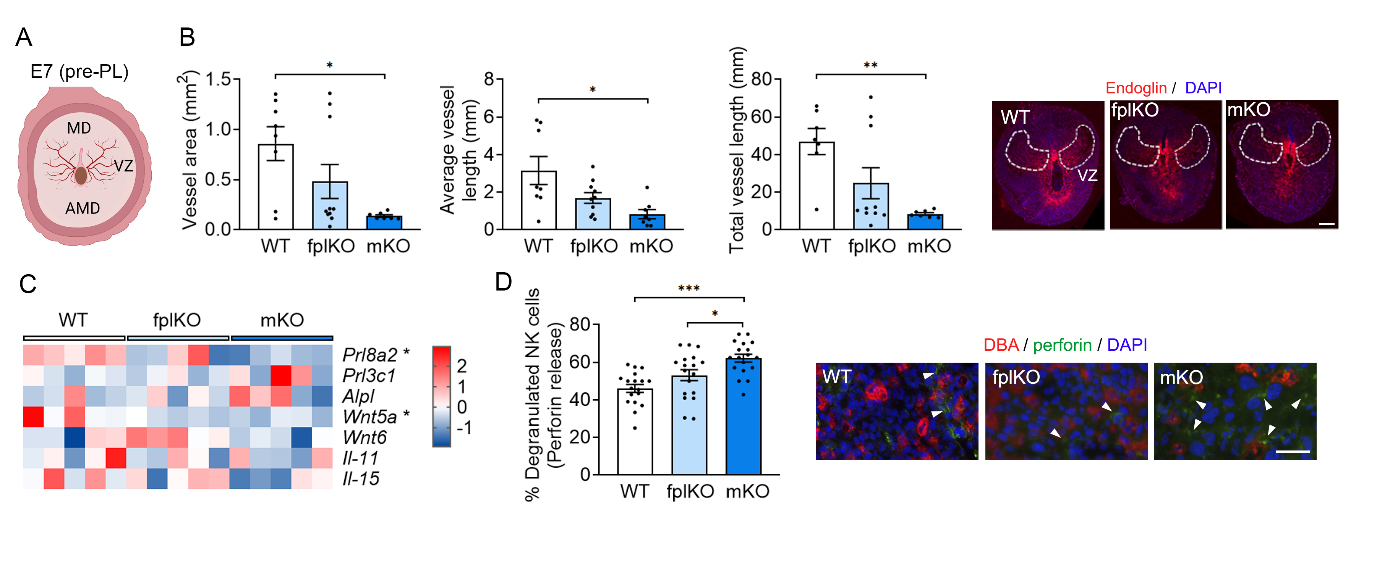


**Fig. S3.** Maternal gal-1 deficiency affects the process of decidualization in mice. A) Schematic representation of implantation site on E7 during the pre-placentation (pre-PL) period. Abbreviations: mesometrial decidua (MD), vascular zone (VZ) and anti-mesometrial decidua (AMD). B) Quantitative results of endoglin expression on the vascular zone (VZ, dotted lines) as analyzed by AngioTool including average vessel length, vessel area, and total vessel length (n=6/ group). Scale bar: 500 µm. *C*) Heatmap showing the expression of genes involved in decidualization in E7 implantation sites analyzed by qPCR followed by Z-score normalization (n=5/ group). Abbreviations: prolactin family 8, subfamily a, member 2 (*Prl8a2*), prolactin family 3, subfamily C, member 1 (*Prl3c1*), alkaline phosphatase (*Alpl*), Wnt family member 5a (*Wnt5a*), Wnt family member 6 (Wnt6), interleukin 11 (*Il-11*) and interleukin 15 (*Il-15*). D) Accumulation of mature uNK cells (DBA^+^) were significantly elevated at E7 and their perforin granules (arrows) were released in mKO dams (n = 5/ group). Scale bar: 50 μm. In all figures, data are expressed mean ± SEM. In Fig. S3B and S3C **P*<0.05 and ***P*<0.01 mKO vs WT using Kruskal-Wallis followed by Dunn’s test and in Fig. S3D **P*<0.05 and ****P*<0.001 using one-way ANOVA followed by Tukey’s test. WT, *Lgals1^+/+^*; fplKO, gal-1 fetal-placental deficiency mice; mKO, gal-1 maternal deficiency mice.

**
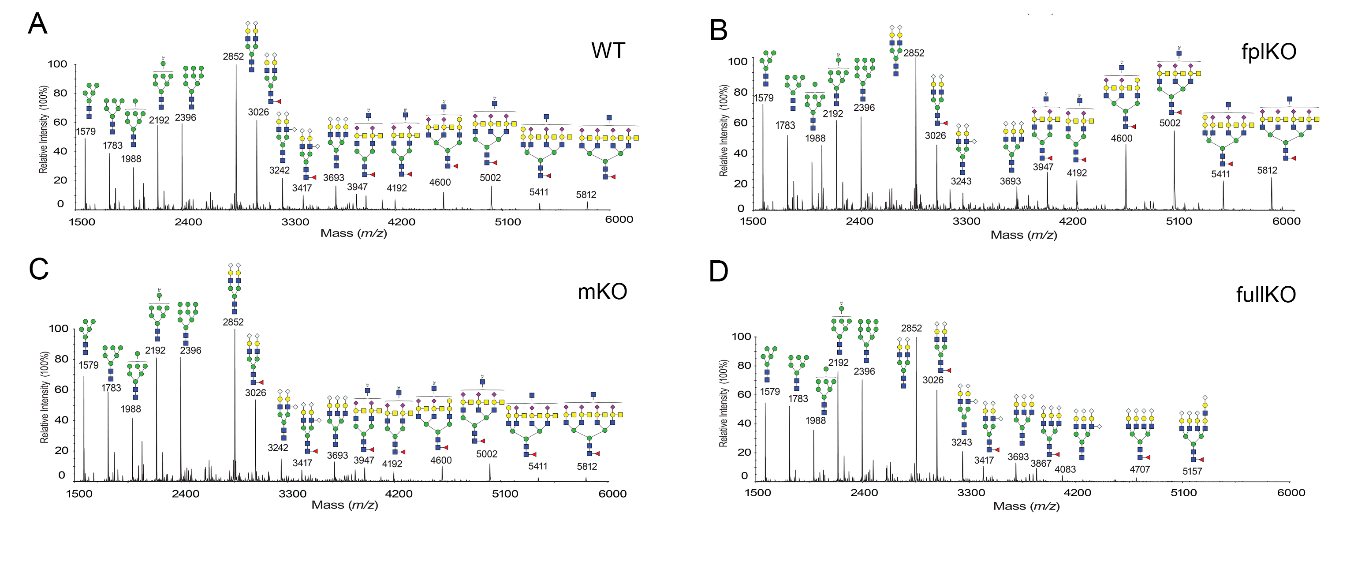
**

**Fig. S4.** MALDI-TOF MS spectra of permethylated N-glycans extracted from murine placental tissue in the mass range 1500 to 6000. Each panel refers to the N-glycan profile established from different galectin-1 knock-out mice placental samples: WT (A), fplKO (B), mKO (C) and fullKO (D). N-glycans were released by N-glycosidase F digestion and permethylated. They were cleaned using a C18 Sep-Pak column. The data presented here are from the glycans that eluted in the 50% acetonitrile fraction. Data were obtained in the positive ion mode, and all ions are sodiated (Na^+^). Structures were determined based on their predicted molecular weight, using knowledge of N-glycans biosynthetic pathways and MS/MS data. WT, *Lgals1^+/+^*; fplKO, gal-1 fetal-placental deficiency mice; mKO, gal-1 maternal deficiency mice; fullKO, *Lgals1^-^*^/-^.


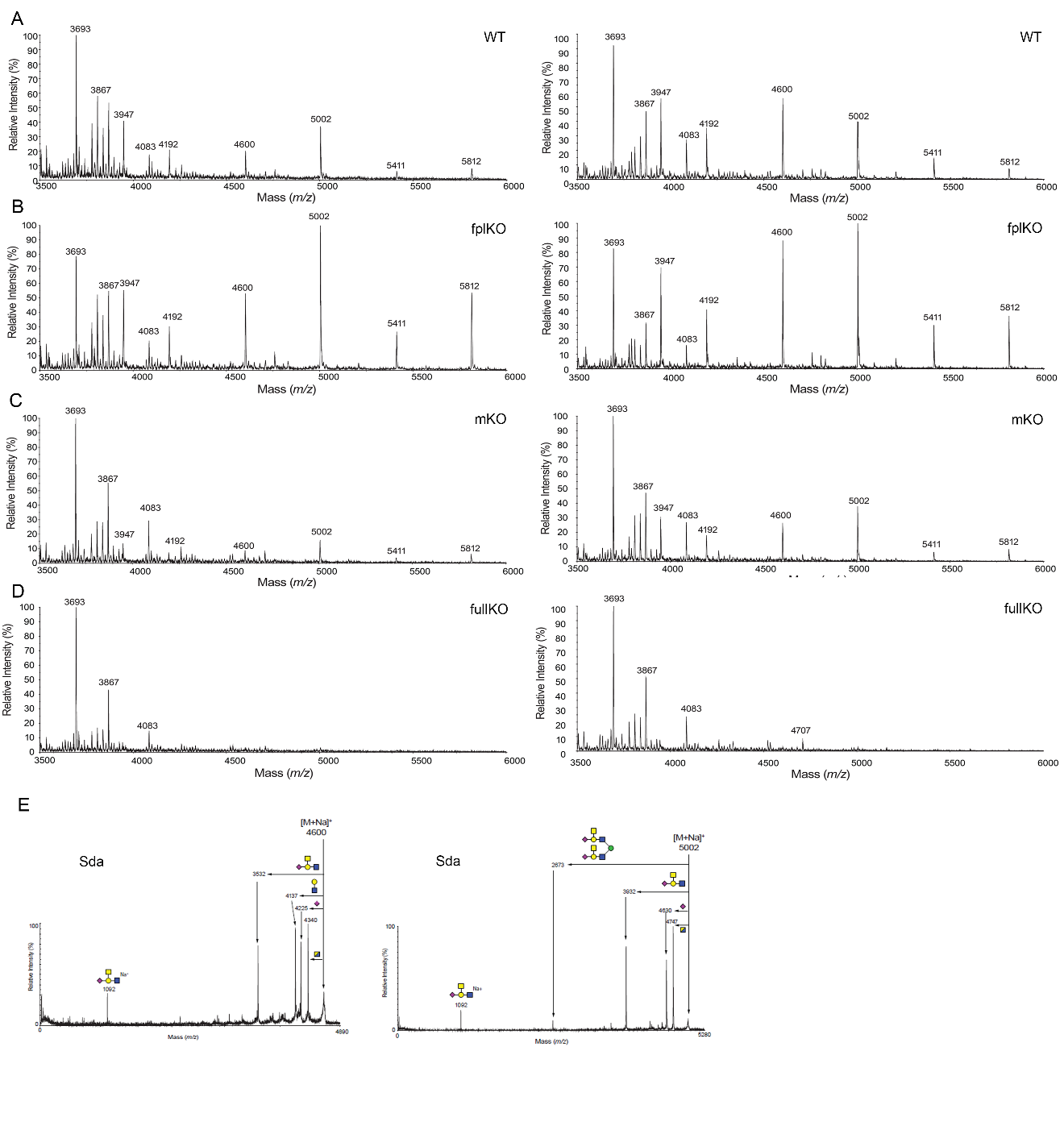


**Fig. S5.** Glycomic profiles of biological replicates generated by MALDI-TOF MS spectra of N-glycans in the high mass range (m/z 3500-6000) extracted from placental tissue of WT (A), fplKO (B) mKO (C) or fullKO (D) mice. E) MALDI-TOF/TOF MS/MS spectra of WT mice for Sda-containing glycans at signals m/z 4600 and 5002. Data were obtained in the positive ion mode. WT, *Lgals1^+/+^*; fplKO, gal-1 fetal-placental deficiency mice; mKO, gal-1 maternal deficiency mice; fullKO, *Lgals1^-^*^/-^.

**
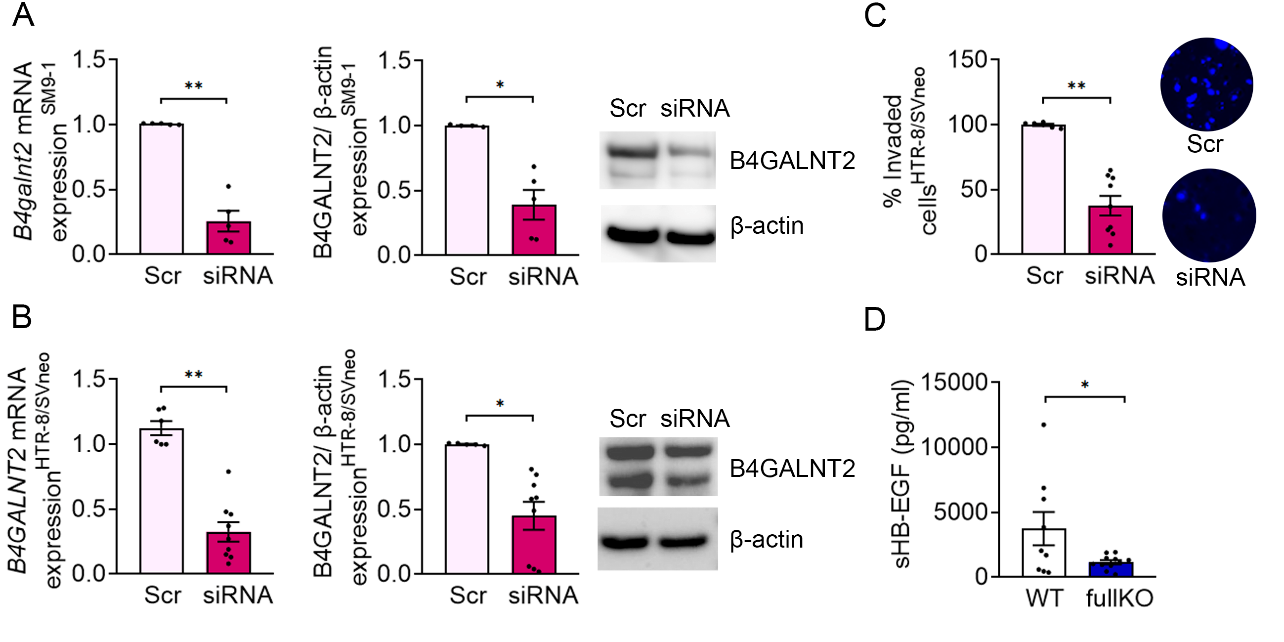
Fig. S6.** Knockdown of endogenous B4GALNT2 in the trophoblast cell lines. B4GALNT2 mRNA/ protein expression is down regulated in treated SM9-1 (A) or HTR-8/SVneo (B) cell lines with small interfering RNA (siRNA) for 36 h, whereas a scramble sequence (Scr) had no effect. Results are expressed as the mean ± SEM of five independent experiments as analyzed by the Mann–Whitney U test, ***P<*0.01 and **P*<0.05. C) The invasion capacity of HTR-8/SVneo cells was evaluated by Transwell invasion assay. Percentage of invaded cells (*left*) and representative images (*right*) of HTR-8/SVneo with siRNA for *B4GALNT2* (siRNA) or a scramble sequence (Scr) as control (n=4, 5 pictures/ assay), ***P*<0.01 using two-tailed *t* test. D) sHB-EGF levels in primary trophoblast cells on E13 as measured by ELISA. sHB-EGF levels from *Lgals1^+/+^* (WT) derived trophoblast cells were increased compared to *Lgals1^-/-^* (fullKO) counterparts. Results are plotted as mean ± SEM, **P*<0.05 using two-tailed *t* test.

**Table S1.** Mouse qPCR primer sequences

| Gene | Forward sequence (5′–3′) | Reverse sequence (5′–3′) |
| --- | --- | --- |
| *Prl8a2* | AGCCAGAAATCACTGCCACT | TGATCCATGCACCCATAAAA |
| *Prl3c1* | GCCACACGATATGACCGGAA | GGTTTGGCACATCTTGGTGTT |
| *Alpl* | CATATAACACCAACGCTCAG | TGGATGTGACCTCATTGC |
| *Wnt5a* | ACGAGGAGCCATGTTCAGAA | ACGCAGGAGGATAACAACCA |
| *Wnt6* | TCCACCTGTTACCAAGGCAT | GGGACCACAAGTTCTCGAGA |
| *Il-11* | CTGACGGAGATCACAGTCTGGA | GGACATCAAGTCTACTCGAAGCC |
| *Il-15* | GTAGGTCTCCCTAAAACAGAGGC | TCCAGGAGAAAGCAGTTCATTGC |
| *Hand-1* | ATCATCACCACTCACACCCG | CTCTGGAAGTAAGGCCGCTC |
| *Prl2c2* | AGCCAGGCTCACACACTATT | ACTAGATCGTCCAGAGGGCT |
| *Prl3d1* | GGCCGCAGATGTGTATAGGG | AGTTTCGTGGACTTCCTCTCG |
| *Ascl2* | GTGAAGGTGCAAACGTCCAC | CCCTGCTACGAGTTCTGGTG |
| *Tpbpa* | GCCAGTTGTTGATGACCCTGA | GCTGTCCATGTTACTGTGGCT |
| *Junb* | AGGCAGCTACTTTTCGGGTC | TTGCTGTTGGGGACGATCAA |
| *Gab1* | ATTTCCACCGTGGATTTGAAC | GATCTATCGCTCGGAAAGGTC |
| *Gcm1* | AAGCTTATTCCCTGCCGAGG | AAAGATGAAGCGTCCGTCGT |
| *B4galnt2* | GCCAGATGCTCCAGTCTATGAG | TCAGGACCTTCCGATGTCTGGT |
| *Gapdh* | TGACGTGCCGCCTGGAGAAA | AGTGTAGCCCAAGATGCCCTTCAG |

**Table S2.** Human qPCR primer sequences

| Gene | Forward sequence (5′–3′) | Reverse sequence (5′–3′) |
| --- | --- | --- |
| *B4GALNT2* | GAGTATTACCCAGACTTGACCG | GTTCCTACCAGCAAACCAAC |
| *GAPDH* | GAAGGTGAAGGTCGGAGTCAA | GGAAGATGGTGATGGGATTTC |

**SI References**

1. Borowski S*, et al.* (2022) Examination of the Contributions of Maternal/Placental-Derived Galectin-1 to Pregnancy Outcome. *Methods Mol Biol* 2442:603-619.

2. Blois SM*, et al.* (2007) A pivotal role for galectin-1 in fetomaternal tolerance. *Nat Med* 13(12):1450-1457.

3. Theiler K (1989) *The House Mouse: Atlas of Embryonic Development.* (New York: Springer-Verlag, Havard.).

4. Freitag N*, et al.* (2013) Interfering with Gal-1-mediated angiogenesis contributes to the pathogenesis of preeclampsia. *Proc Natl Acad Sci U S A* 110(28):11451-11456.

5. Schwedhelm E*, et al.* (2007) High-throughput liquid chromatographic-tandem mass spectrometric determination of arginine and dimethylated arginine derivatives in human and mouse plasma. *J Chromatogr B Analyt Technol Biomed Life Sci* 851:211-219.

6. Freitag N*, et al.* (2014) Influence of relative NK-DC abundance on placentation and its relation to epigenetic programming in the offspring. *Cell Death Dis* 5(8):e1392.

7. North SJ*, et al.* (2010) Mass spectrometric analysis of mutant mice. *Methods Enzymol* 478:27-77.

8. Pennington KA, Schlitt JM, & Schulz LC (2012) Isolation of primary mouse trophoblast cells and trophoblast invasion assay. *Journal of visualized experiments : JoVE* (59):e3202.

9. Kieckbusch J, Gaynor LM, & Colucci F (2015) Assessment of Maternal Vascular Remodeling During Pregnancy in the Mouse Uterus. *Journal of visualized experiments : JoVE* (106):e53534.

10. Zhang JH, Yamada AT, & Croy BA (2009) DBA-lectin reactivity defines natural killer cells that have homed to mouse decidua. *Placenta* 30(11):968-973.

11. Zudaire E, Gambardella L, Kurcz C, & Vermeren S (2011) A computational tool for quantitative analysis of vascular networks. *PLoS One* 6(11):e27385.

12. Ceroni A*, et al.* (2008) GlycoWorkbench: a tool for the computer-assisted annotation of mass spectra of glycans. *J Proteome Res* 7(4):1650-1659.

13. Budwit-Novotny DA*, et al.* (1986) Immunohistochemical analyses of estrogen receptor in endometrial adenocarcinoma using a monoclonal antibody. *Cancer Res* 46(10):5419-5425.
